# Detection of ribonucleotides embedded in DNA by Nanopore sequencing

**DOI:** 10.1038/s42003-024-06077-w

**Published:** 2024-04-23

**Authors:** Lavinia Grasso, Adriano Fonzino, Caterina Manzari, Tommaso Leonardi, Ernesto Picardi, Carmela Gissi, Federico Lazzaro, Graziano Pesole, Marco Muzi-Falconi

**Affiliations:** 1 Dipartimento di Bioscienze, Università degli Studi di Milano, Via Celoria 26, 20133 Milano, Italy; 2 Dipartimento di Bioscienze, Biotecnologie e Ambiente, Università di Bari A. Moro, Via Orabona 4, 70126 Bari, Italy; 3 Center for Genomic Science of IIT@SEMM, Fondazione Istituto Italiano di Tecnologia, Via Adamello 16, 20139 Milano, Italy; 4 Istituto di Biomembrane, Bioenergetica e Biotecnologie Molecolari, Consiglio Nazionale delle Ricerche, Via Amendola 122/O, 70126 Bari, Italy

**Keywords:** Molecular biology, Computational biology and bioinformatics

## Abstract

Ribonucleotides represent the most common non-canonical nucleotides found in eukaryotic genomes. The sources of chromosome-embedded ribonucleotides and the mechanisms by which unrepaired rNMPs trigger genome instability and human pathologies are not fully understood. The available sequencing technologies only allow to indirectly deduce the genomic location of rNMPs. Oxford Nanopore Technologies (ONT) may overcome such limitation, revealing the sites of rNMPs incorporation in genomic DNA directly from raw sequencing signals. We synthesized two types of DNA molecules containing rNMPs at known or random positions and we developed data analysis pipelines for DNA-embedded ribonucleotides detection by ONT. We report that ONT can identify all four ribonucleotides incorporated in DNA by capturing rNMPs-specific alterations in nucleotide alignment features, current intensity, and dwell time. We propose that ONT may be successfully employed to directly map rNMPs in genomic DNA and we suggest a strategy to build an ad hoc basecaller to analyse native genomes.

## Abstract (web summary)

This study reports the direct detection of ribonucleotides embedded in DNA molecules by Oxford Nanopore sequencing, based on ribonucleotides-induced alterations in basecalling features, currents, and dwell times.

## Introduction

Due to the inherent chemical instability of RNA molecules, living organisms usually store their genetic information in DNA. DNA, indeed, lacks the reactive 2′-OH group of the ribose ring, which can attack the sugar-phosphate backbone, generating breaks with genotoxic outcomes^1^. To guarantee the proper transmission of genetic information, cells must duplicate their DNA extremely faithfully, avoiding mutations that can promote genome instability, leading to pathologies like cancer and neurodegenerative diseases^2,3^. Nevertheless, DNA integrity is constantly challenged by a variety of exogenous and endogenous sources of damage and replication stress^4–6^.

Ribonucleotides represent the most common non-canonical nucleotides found in the eukaryotic genome^7–11^. Single rNMPs insertions in the genome primarily result from the ability of replicative DNA polymerases to duplicate chromosomal DNA, despite their high-fidelity rates^7,8,10,12^. Other cellular processes potentially contributing to the incorporation of ribonucleotides in DNA are Okazaki fragments priming, R-loops formation and reparative DNA synthesis^13^. The abundance and, to a certain degree, biassed distribution of ribonucleotides in the eukaryotic genome^14–16^ implies a biological relevance in specific cellular contexts. For example, it was demonstrated that chromosome-embedded rNMPs provide sites of incision to initiate the mismatch repair (MMR) pathway in the leading strand of DNA^17,18^. Although having physiological functions, ribonucleotides must be only transiently present in the genome and their prompt removal from DNA is fundamental to prevent negative consequences^19–21^. Unrepaired ribonucleotides, especially in stretches of multiple insertions, can affect the structure of the DNA double helix^22–25^ and the assembly of nucleosomes^26,27^, they can hamper the progression of the replicative DNA polymerases^10,12,28^ and they can favour mutagenesis and genomic instabilities^29–31^. To restore the correct DNA:DNA composition, cells are equipped with ribonucleases H (RNase H1 and H2 in eukaryotes), endonucleolytic enzymes specialised in the removal of ribonucleotides from double-stranded (dsDNA) molecules^32^. RNase H2 mutations are associated with Aicardi–Goutières syndrome (AGS)^33^, a rare autoinflammatory disorder that mainly affects the brain, and patient-derived cells show an accumulation of rNMPs in their genome^34–36^. RNase H2 dysfunctions have also been linked to some cancers^37–41^ and to systemic lupus erythematosus (SLE)^42^.

In order to fully unravel the mechanisms responsible for embedding rNMPs in chromosomal DNA, to define the molecular details of ribonucleotide-induced genome instability and to determine the detrimental impact that DNA-embedded ribonucleotides have on cells and patients, it is crucial to identify exactly how and where rNMPs are localised in the genome. Different high-throughput sequencing techniques have been developed to map ribonucleotides at the genomic level with single-nucleotide resolution^14,16,43–47^. All these methods are based on the generation of breaks in genomic DNA by either enzymatic or chemical digestion at the DNA-RNA junction. Hence, they share some limitations: they only allow to indirectly deduce the location of ribonucleotides in the genome, and they fail to eventually discriminate between a single rNMP or a potentially more harmful stretch of consecutive rNMPs at a certain position^13^. Additionally, they were applied to constitutively/permanently RNase H-deficient yeast strains, which accumulate thousands of ribonucleotides in the template DNA. As mentioned above, this can compromise the progression and fidelity of the replicative DNA polymerases^10,12,28^, conceivably altering the real sites of rNMPs incorporation in a single round of DNA replication.

Direct sequencing would be the best approach to identify the location of single and multiple consecutive rNMPs in genomic DNA. In this context, the sequencing technologies developed by Oxford Nanopore Technologies (ONT) may provide an appealing solution. Nanopore sequencing (extensively reviewed in^48–50^) is based on the use of engineered nanopores serving as biosensors embedded in a membrane of electrically resistant synthetic polymers. A voltage bias of about 200 mV is usually clamped across the two sides of the membrane so that ions in an electrolytic solution flow freely through the pores, generating an open current that is measured over time. A motor protein with helicase activity enables dsDNA or RNA:DNA duplexes to unwind and controls the translocation of single-stranded DNA or RNA molecules through the nanopore from the negatively to the positively charged side at a translocation speed ranging from 260 to 520 bp/s for DNA strands. Each nucleotide crossing the sensing region generates a characteristic disruption in the ion flow, detectable as a distinct change in the open current. Nucleotide identity is decoded using specific machine learning-based algorithms, allowing real-time sequencing of single molecules. Nanopore sequencing sensitivity and versatility not only allow for the detection of the four canonical bases in a nucleic acid filament, but it also permits the identification of base analogues^51–53^, nucleotide modifications as tiny as methylation^52^, and other structures like DNA adducts^54,55^ or non-B DNA structures (as G-quadruplexes)^56^. These features make Nanopore sequencing the perfect candidate tool to attempt to directly identify and map rNMPs embedded in genomic DNA.

In this study, we assessed the feasibility of directly detecting rNMPs embedded in DNA molecules by Nanopore sequencing. We sequenced and analysed two types of rNMPs-containing synthetic DNA molecules and we developed dedicated bioinformatic tools for their recognition. First, by using rNMPs-containing synthetic DNA primers complementary to the viral M13mp18 circular single-stranded DNA (ssDNA), we generated linear dsDNA fragments containing single rNMPs at known positions. Second, taking advantage of a *Taq*-I614K DNA polymerase mutant produced in our laboratory^25,57^, we obtained, from the same template, dsDNA molecules with randomly incorporated rNMPs. Nanopore libraries were constructed without prior chemical processing of the ribonucleotides so that rNMPs were in their unmodified native state. In-house ad hoc data analysis pipelines based on the assessment of nucleotide alignment errors together with alterations in reference-anchored current intensity values and dwell times were successfully elaborated and exploited, following the approach used for the detection of other nucleotide modifications by the ONT sequencing platform^58–62^. Our results show an unexplored ability of Nanopore sequencing not only to identify the occurrence of all four ribonucleotides incorporated in DNA molecules at known positions but also to recognise sites where rNMPs were randomly incorporated in DNA. These findings demonstrate that Nanopore sequencing may successfully be employed to directly detect and map rNMPs embedded in native genomic DNA. Moreover, we identified the tools that may be exploited to build a specialised basecaller able to reveal the presence and exact positions of rNMPs in chromosomal DNA.

## Results

### Construction and sequencing of synthetic dsDNA substrates containing single rNMPs at known positions

**Fig. 1:**
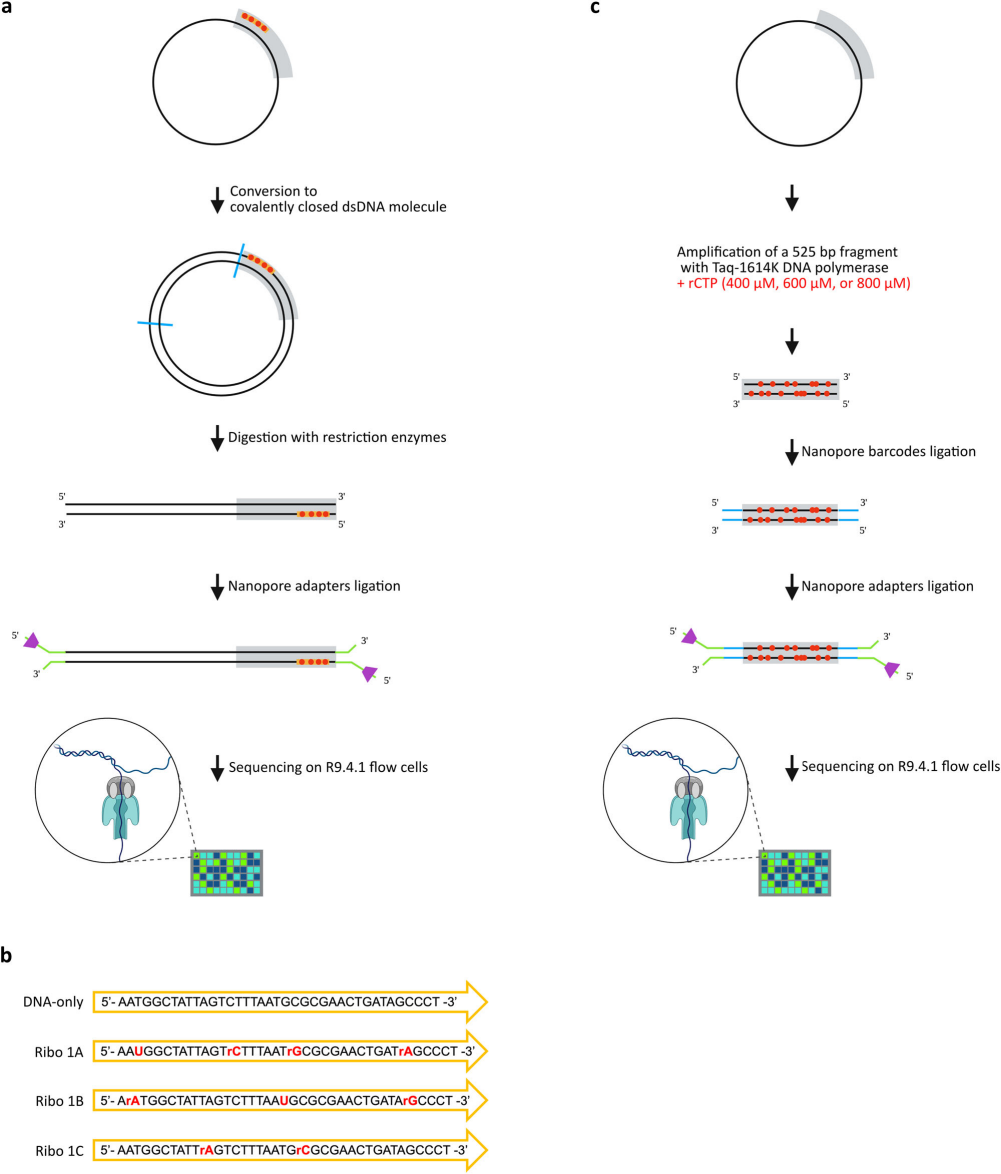
Construction and sequencing of synthetic dsDNA molecules containing rNMPs. **a** Scheme of the procedure followed to obtain dsDNA substrates with rNMPs at known positions. An in vitro extension reaction was carried out using the viral M13mp18 circular ssDNA as a template and a complementary DNA oligonucleotide containing single rNMPs (red dots) as a primer. The generated circular dsDNA was enzymatically treated to get covalently closed molecules and cleaved with restriction enzymes (blue lines). Nanopore adapters were ligated to the ends of the resulting 5545 bp-long fragments containing rNMPs at known positions. Nanopore libraries were sequenced on MiniION R9.4.1 flow cells. **b** Primers pairing to the same region on the M13mp18 circular ssDNA were employed to independently generate a “DNA only” control library and three different libraries with rNMPs at known positions: “Ribo1A”, “Ribo1B” and “Ribo1C” (black dNMPs; red rNMPs). **c** Scheme of the procedure followed to obtain dsDNA substrates with randomly incorporated rNMPs. 525 bp-long fragments were PCR-amplified from the viral M13mp18 circular ssDNA by exploiting the *Taq*-I614K DNA polymerase that randomly incorporates rCMPs (red dots). The generated fragments containing rCMPs at unknown positions were sequentially ligated to Nanopore barcodes and adapters. Nanopore libraries were sequenced on MiniION R9.4.1 flow cells. Grey boxes indicate the sequence in common between the two types of rNMPs-containing synthetic substrates.

To determine whether Nanopore sequencing technologies are suitable for direct detection of ribonucleotides embedded in DNA, we needed special DNA substrates containing the four rNMPs at known positions so that, if a specific sequencing signal was detected, it could be traced back to the presence of a specific rNMP. To this aim, we designed DNA oligonucleotides containing single rNMPs, complementary to the viral M13mp18 circular ssDNA, which we exploited as primers and templates, respectively, for in vitro extension reactions (Fig. 1a). In particular, we designed three distinct oligonucleotides (Ribo1A, Ribo1B and Ribo1C) complementary to the same M13mp18 region, each containing different combinations of 2–4 single rNMPs embedded in different DNA sequence contexts, for a total of nine single rNMP substitutions (Fig. 1b). These oligonucleotides were used to perform three independent in vitro extension reactions. The integrity of the generated M13mp18 circular dsDNA was ensured by enzymatic treatment to obtain covalently closed molecules that were cleaved with a combination of restriction enzymes to produce linear dsDNA fragments (Fig. 1a). Such synthetic dsDNA substrates containing rNMPs in the proximity of the 5’ end of the strand complementary to the original M13mp18 ssDNA template, were ligated to Nanopore adapters (Fig. 1a), according to the standard Nanopore library construction protocol. The resulting “Ribo1A”, “Ribo1B” and “Ribo1C” libraries were sequenced on R9.4.1 flow cells (Fig. 1a). A “DNA-only” control library of dsDNA fragments having the same sequence of the rNMPs-containing dsDNA fragments, but without rNMPs, was obtained with the same procedure, using an oligonucleotide entirely made of dNMPs. A total amount of reads of 1.86E + 05 for “DNA-only”, 5.34E + 05 for “Ribo1A”, 5.75E + 06 for “Ribo1B” and 4.81E + 06 for “Ribo1C” passed the quality control checks and were successfully basecalled by the Guppy basecaller.

### Ribonucleotides embedded in DNA at known positions can be identified by basecalling errors and by alterations in current intensity and dwell time profiles

Current intensity signals generated by Nanopore sequencing are recorded in real-time with a sampling frequency of 4 kHz and provide information about the 5- to 6-mer sequence context inside the pocket of the pore at a given time *t*^58,61,63–65^. These signals are stored as time-series data in fast5 files and need to be translated into nucleotide sequences to allow downstream analysis, such as basic alignment to a reference sequence. This conversion is a very demanding computational problem, as the same nucleotide can be responsible for completely different current intensity signals depending on the surrounding sequence context. The process of translating raw ONT data into nucleotide sequences is known as “basecalling”, and it is presently achieved through sequence-to-sequence deep neural network (DNN) algorithms, even though it represents a continuously evolving research field. Since specific nucleotide modifications in both DNA and RNA alter the raw signal generated by the sequencing machinery in a peculiar manner, they can be detected either by expanding the basecaller vocabulary with additional nucleotides or by searching for systematic, reproducible and non-random “errors” in basecalling features and nucleotide alignment profiles, probably due to misinterpretations of the signal deriving from a modified nucleotide that is not included in the basecaller training dataset^52,58,63^.

**Fig. 2:**
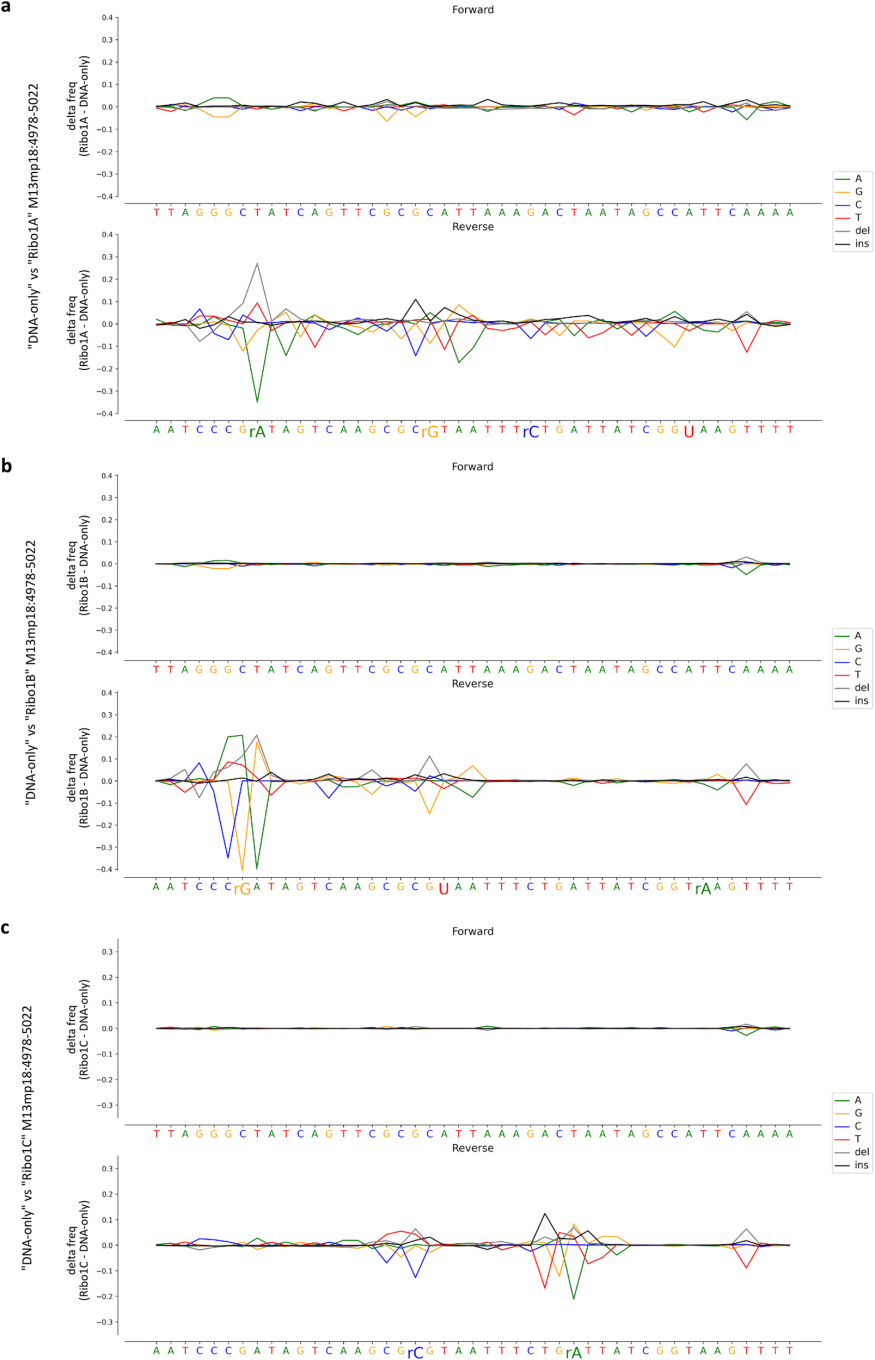
Detection of rNMPs at known positions by nucleotide sequence alignment profiles analysis. BAM files were split into forward and reverse strands, and nucleotide sequences were independently inferred from the “DNA only” and the three “Ribo” runs. The difference in the frequency of detection of A (green), G (yellow), C (blue), T (red), deletions (grey) and insertions (black) was then calculated at each position of each “Ribo” sample compared to the “DNA only” control. Each panel shows the nucleotide sequence alignment profiles of the forward (upper graphs) and reverse (bottom graphs) strands for M13mp18 genomic coordinates from 4978 to 5022 that include the entire region where control and rNMPs-containing primers were annealed. Single rNMPs insertions are expected on the reverse strand. **a** “DNA-only” vs “Ribo1A” runs, **b** “DNA-only” vs “Ribo1B” runs, **c** “DNA-only” vs “Ribo1C” runs. Alignment profiles are depicted in the 5′–3′ direction on the forward strand, so the complementary sequence is indicated for the reverse plots.

Based on these assumptions, we initially attempted to recognise DNA-embedded ribonucleotides from alterations in the nucleotide alignment profiles. As indicated in the workflow summary of Supplementary Fig. 1, the fast5 files generated during sequencing were converted to fastQ files and aligned to the M13mp18 ssDNA reference sequence by minimap2. The generated BAM files were split into forward (+) strand sequences, directly mapping to the M13mp18 ssDNA sequence, and reverse (−) strand sequences, mapping to the sequence complementary to the M13mp18 ssDNA and expected to contain the single rNMP substitutions in the primer region. Nucleotide sequences were independently retrieved from “DNA-only”, “Ribo1A”, “Ribo1B” and “Ribo1C” runs. For the “DNA-only” run, 83.44% of the passed reads were successfully mapped against the reference sequence by the aligner, of which 46.77% were mapped on the reverse (-) strand. An average of 91.72% of the passed reads of the three “Ribo” runs were aligned to the reference sequence, of which an average of 49.96% mapped on the reverse (−) strand. The nucleotide alignment profiles of forward and reverse strands were separately obtained by plotting the difference in the frequency of detection of A, C, G, T, deletions, and insertions measured at each M13mp18 genomic coordinate for each “Ribo” sample respect to “DNA-only” (Fig. 2). Strikingly, when comparing each “Ribo” sample to the control, numerous, reproducible alterations resulting in a noisier alignment profile, were detected only on the reverse strand in correspondence of the regions known to contain ribonucleotides (Fig. 2a–c, bottom graphs). On the other hand, no clear and reproducible alterations were detected on the forward strand (Fig. 2a–c, upper graphs). According to our experimental design, we expected four rNMP substitutions on the reverse strand of the “Ribo1A” sample (rA at position 4985, rG at position 4997, rC at position 5004 and U at position 5015), three rNMP substitutions on the reverse strand of the “Ribo1B” sample (rG at position 4984, U at position 4998 and rA at position 5016), and two rNMP substitutions on the reverse strand of the “Ribo1C” sample (rC at position 4996, and rA at position 5007) (Fig. 1b). At all these locations, indeed, we generally observed a decrease in the frequency of detection of the predicted base, compensated by an increase in the rate of mismatches or indels (Fig. 2a–c, bottom graphs). Interestingly, similar perturbations were observed not only in correspondence of the exact coordinates where ribonucleotides were included in the “Ribo” primers but also at some nearby positions (Fig. 2a–c, bottom graphs). This is consistent with the fact that, as already pointed out, ONT electric signals, and thus basecalling features, depend on a stretch of 5–6 nucleotides passing through nanopore channels at a given time. A single rNMP is, therefore, presumed to affect the signals generated by the surrounding nucleotides. The alterations due to the presence of rA, except for rA at position 5016, and rG were generally more evident, while the ones induced by rC and U were less pronounced but still clearly visible (Fig. 2a–c, bottom graphs). In summary, we demonstrated that the presence of all four rNMPs embedded in DNA caused an increased rate of basecalling errors not only in correspondence of the ribonucleotide itself but also in the immediately surrounding area.

Many computational tools available for the detection of base modifications by ONT use current intensity-based methods^59,60,62,66,67^. The starting point of every ONT sequencing experiment is, indeed, constituted by the electric signals stored inside the fast5 files generated by the sequencer. When the final goal is to look for dissimilarities between different samples, as in our case, an effective approach is to directly investigate the current intensity signals.

**Fig. 3:**
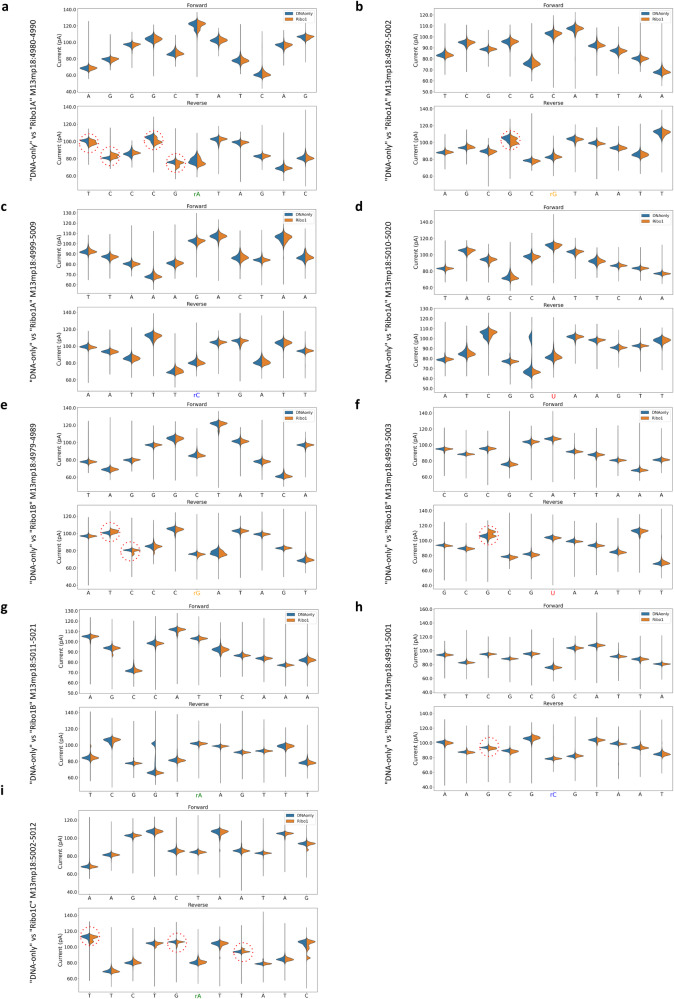
Detection of rNMPs at known positions by current intensity profiles analysis. The f5c eventalign software was used to align current intensities back to the M13mp18 reference genome sequence, with the aim to compare mean current intensities at each genomic coordinate of each “Ribo” sample (orange) to the “DNA only” (blue) control. Each panel shows a subset of 11 positions on the forward (upper graphs) and reverse (bottom graphs) strands, centred on each genomic coordinate where single rNMPs were inserted on the reverse strand of the “Ribo” samples: rA at position 4985 (**a**), rG at position 4997 (**b**), rC at position 5004 (**c**) and U at position 5015 (**d**) in “Ribo1A”; rG at position 4984 (**e**), U at position 4998 (**f**) and rA at position 5016 (**g**) in “Ribo1B”; rC at position 4996 (**h**) and rA at position 5007 (**i**) in “Ribo1C”. Ionic current profiles are depicted in the 5′–3′ direction on the forward strand, so the complementary sequence is indicated for the reverse plots. The positions where the “Ribo” samples show a clear bimodal distribution on the reverse strand, not observable for the “DNA-only” control, are highlighted by dotted, red circles.

Therefore, in addition to exploiting mistakes in basecalling and nucleotide alignment profiles, we opted for comparing the current intensity profiles of each “Ribo” run to the one of the “DNA-only” control. A preliminary step for the analysis of current intensity profiles consists in anchoring the electric events contained in the raw data files to the reference genome. As reported in the workflow summary of Supplementary Fig. 1, the BAM files generated by minimap2 were, thus, used for “re-squiggling” the electric events against the M13mp18 reference sequence with the f5c eventalign software in order to compare the forward and reverse current distributions derived from “Ribo1A”, “Ribo1B” and “Ribo1C” samples to the ones derived from the “DNA-only” sample. Comparably to what we found out from the analysis of nucleotide alignment profiles, numerous, reproducible current intensity alterations were clearly visible only on the reverse strand around the regions containing the 9 single ribonucleotides (Fig. 3a–i, bottom graphs). In this case, the strongest perturbations consisted in some positions of the “Ribo” samples showing bimodal distributions of the current, not noticeable for the corresponding positions of the “DNA-only” control (Fig. 3a, b, e, f, h, i, bottom graphs, dotted, red circles). Again, these alterations were not limited to the genomic coordinate where each rNMP was included, but they were extended to the surrounding nucleotides. As expected, no clear signs of alteration were detected on the forward strand (Fig. 3a–i, upper graphs) when comparing “Ribo1A”, “Ribo1B and “Ribo1C” runs to the “DNA-only” control. The presence of all these electric signal changes only on the reverse strand confirmed that we were really observing perturbations caused by the presence of ribonucleotides in DNA.

Another strategy used for direct detection of nucleotide modifications by Nanopore sequencing is based on the analysis of the dwell time^68,69^, which is the time a nucleotide spends inside the nanopore channel during sequencing.

**Fig. 4:**
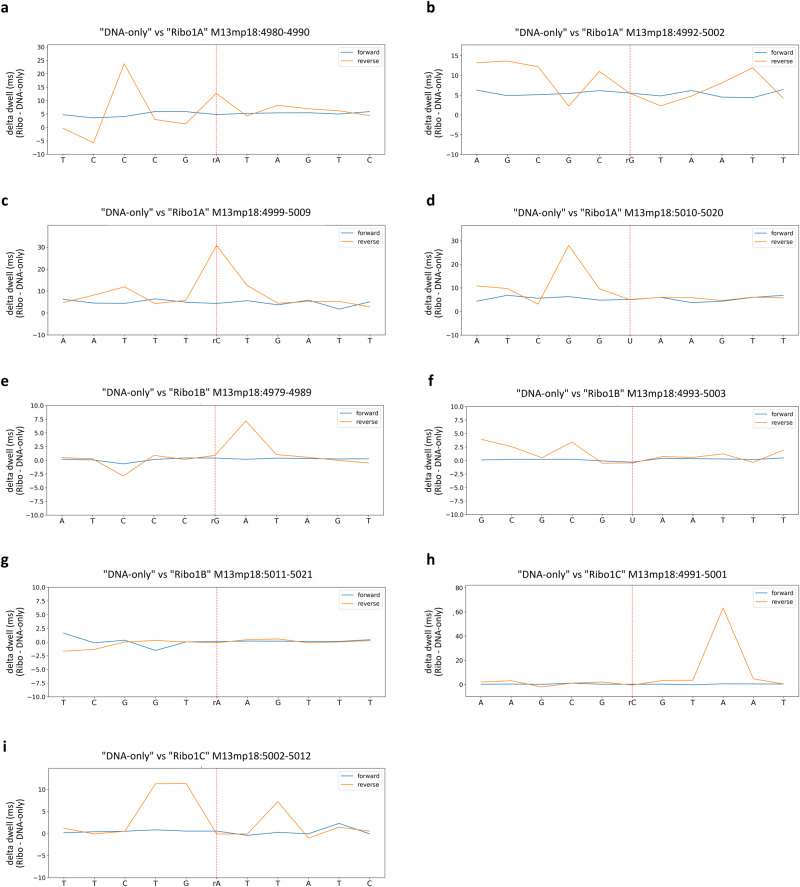
Detection of rNMPs at known positions by dwell time profiles analysis. Dwell times were extracted using the f5c eventalign software. Each panel shows the delta in the mean dwell time values retrieved from the two experimental conditions (“Ribo”–“DNA only”) and separately calculated at each position of the forward (blue) and reverse (orange) strands. Each panel represents a subset of 11 positions on the forward (upper graphs) and reverse (bottom graphs) strands, centred on each genomic coordinate where single rNMPs were expected on the reverse strand: rA at position 4985 (**a**), rG at position 4997 (**b**), rC at position 5004 (**c**), and U at position 5015 (**d**) in “Ribo1A”; rG at position 4984 (**e**), U at position 4998 (**f**), and rA at position 5016 (**g**) in “Ribo1B”; rC at position 4996 (**h**) and rA at position 5007 (**i**) in “Ribo1C”. Dwell times are depicted in a 5′ to 3′ direction on the forward strand, so the complementary sequence is indicated for the reverse plots.

Dwell times were extracted by exploiting again the f5c eventalign software. The dwell time profiles of the forward and reverse strands were separately obtained by calculating the difference between the mean dwell time value of each “Ribo” run and the mean dwell time value of the “DNA-only” run at each position (Fig. 4). Dwell time profiles analysis of the reverse strand revealed clear alterations related to the presence of all rNMPs embedded in “Ribo1A”, “Ribo1B” and “Ribo1C” samples, except for rA at position 5016 (Fig. 4, orange lines). By contrast, dwell time profiles of the forward strand showed no clear signs of alteration (Fig. 4, blue lines). Once more, these perturbations were not restricted to the genomic coordinates corresponding to each rNMP, but they spanned on several upstream and downstream proximal positions. These findings are in accordance with the observations previously made by analysing nucleotide alignment profiles and current intensities.

Taken together, these results indicate that all four rNMPs embedded in DNA can be successfully identified by Nanopore sequencing by searching for rNMPs-related errors in basecalling, perturbations in current distributions, and dwell time profiles.

### Ribonucleotides embedded in DNA at known positions can be efficiently recognised from a mixture of dNMPs- and rNMPs-containing reads in silico

As mentioned in the introduction, single rNMPs incorporations are mostly caused by replicative DNA polymerase misinsertions^7,8,10,12^. Then, a sample of genomic DNA would not always contain a rNMP at a certain position.

**Fig. 5:**
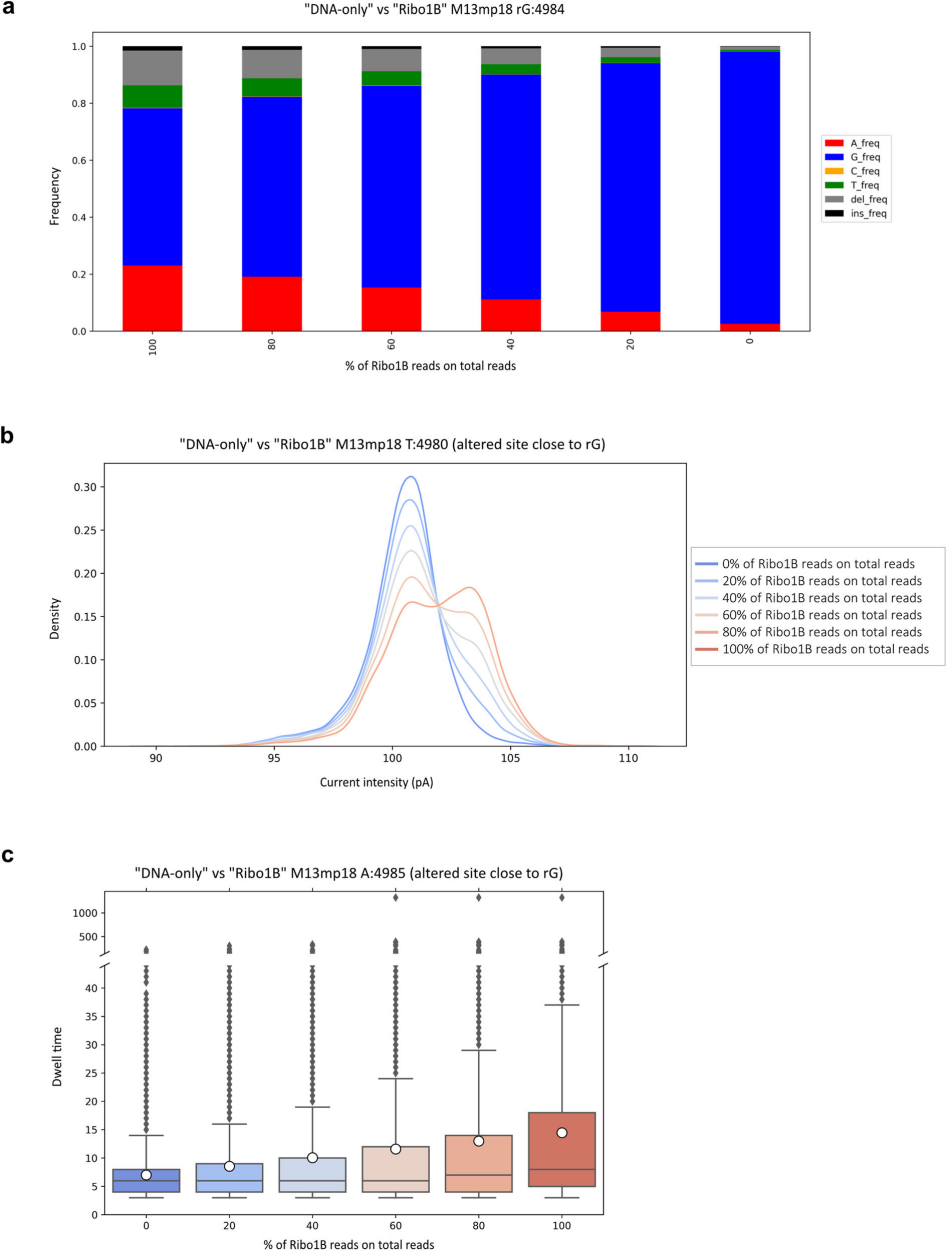
rG can be recognised in a mixture of DNA and ribonucleotide-containing reads. An in silico simulated washout assay was performed as follows on the “DNA-only” and “Ribo1B” runs. A random sample made of a fixed total number of reads mapping on the reverse strand was extracted from “DNA-only” and “Ribo1B” runs and mixed at different ratios of “Ribo1B” derived reads with respect to “DNA-only” control reads. The effect of each ratio was deeply evaluated on altered sites within a ±5 nt long interval surrounding the site of rG incorporation at position 4984. **a** Alterations in the basecalling features of each mixture of reads at position 4984, shown as modifications in the frequency of detection of A (red), G (blue), C (yellow), T (green), insertions (black) and deletions (grey). **b** Alterations in ionic current distributions of events retrieved via f5c eventalign software and re-squiggled on-site T:4980. **c** Alterations in dwell time values measured at site A:4985, shown as a box plot with the median as a solid black line, the mean as a white circle, the first and third quartiles captured by the boundaries of the box, whiskers defined as the first and third quartiles ± interquartile range 1.5 times, and outliers depicted as black points.

We, therefore, wondered whether the signal of a specific rNMP would still be recognisable in a sample that contained molecules with and without rNMPs at a given location. To investigate this, we performed an in silico simulated washout assay (Fig. 5), in which we virtually mixed at different ratios a fixed total number of reads mapping on the reverse strand, randomly extracted from the “DNA-only” control and the “Ribo1B” sample. For each ratio, we evaluated all the previously analysed features around the site of rG incorporation at position 4984.

The analysis of nucleotide alignment profiles of “Ribo1B” compared to “DNA-only” showed that, on the reverse strand, rG:4984 was responsible for very pronounced alterations at its genomic coordinate (Fig. 2b). The analysis of basecalling features at position 4984, indeed, revealed that when reads uniquely derived from the “DNA-only” sample (0% of “Ribo1B” reads on total reads), the vast majority of basecalled events corresponded to G (Fig. 5a). When “Ribo1B” derived reads represented 20% of the total number of reads, the frequency of detection of A and, to a lesser extent, T, deletions and insertions was increased to a total error frequency higher than 10% (Fig. 5a). The frequency of detection of A, T, deletions and insertions kept rising together with the fraction of “Ribo1B” derived reads. At 100% of “Ribo1B” derived reads, the total frequency of errors was higher than 40% (Fig. 5a).

When looking at the current distributions of “Ribo1B” and “DNA-only” on the reverse strand, we observed that rG:4984 generated a peculiar bimodal distribution of the current at position T:4980 (Fig. 3e). For this reason, we evaluated the effect of increasing fractions of “Ribo1B” derived reads on the current intensity at this position. In accordance with what was described above, the effect of rG on the electric signals was detectable when “Ribo1B” derived reads constituted 20% of the total of reads, and it increased with the fraction of “Ribo1B” derived reads (Fig. 5b).

As already shown in Fig. 4e, reproducible dwell time perturbations generated by rG:4984 were observed at coordinate A:4985, when comparing the reverse strands of “Ribo1B” and “DNA-only” runs. The impact of increasing percentages of “Ribo1B” reads on dwell time at that position was consistent with the previous results: dwell time values went up along with the fraction of “Ribo1B” reads on the number of total reads (Fig. 5c).

Interestingly, in silico simulated washout experiments conducted on all the other rNMPs, excluded rA at position 5016 that did not show evident perturbations, led to similar alteration patterns (Supplementary Fig. 2).

In light of these observations, we can conclude that ONT allows the detection of DNA-embedded ribonucleotides even in a sample where, at a given position, dNMP- and rNMP-containing reads are mixed. These analyses were performed in situations in which features were aggregated onto genomic positions. Different strategies tailored to increase the granularity at a per-read level would strongly lower down the minimal percentage of ribonucleotides needed at a certain position for their successful detection, which would be relevant for their visualisation inside genomic DNA samples.

### Construction and sequencing of dsDNA substrates containing randomly incorporated rCMPs

To assess the performance of ONT sequencing on substrates more similar to genomic DNA samples, we generated dsDNA molecules containing rCMPs at unknown, random positions. We took advantage of a *Taq*-I614K DNA Polymerase mutant^25,57^ that was produced in our laboratory and was extensively characterised for its ability to synthesise hundreds-of-bp-long, rCMP-containing DNA molecules in the presence of all four dNTPs and rCTP^25^. *Taq*-I614K DNA Polymerase, although designed to incorporate rCTPs, cannot function in the absence of dCTP during the synthesis; the addition of dCTP to the reaction is thus necessary. Therefore, the incorporation of rCMPs will be random^25^. Using the M13mp18 DNA as a template, we employed *Taq*-I614K to generate dsDNA fragments of 525 bp in the presence of 400, 600, or 800 μM rCTP. Such synthetic dsDNA substrates containing rCMPs in both strands were subsequently ligated to Nanopore barcodes and adapters (Fig. 1c). The resulting Nanopore “rC 400 μM”, “rC 600 μM” and “rC 800 μM” libraries, constituted by dsDNA fragments containing a different combination of rCMP incorporations at unknown positions on both strands, were finally sequenced on R9.4.1 flow cells (Fig. 1c). The same procedure was followed in a PCR reaction in which *Taq*-I614K was not provided with rCMP, to obtain a “DNA-only” control library. Importantly, the 525 bp fragments containing randomly incorporated rCMPs included the region used to produce the DNA fragments with ribonucleotides at known positions described previously. In this way, the two types of rNMPs-containing synthetic dsDNA substrates shared the same sequence (Fig. 1a, c, grey boxes), which would allow us to eventually compare them in the following analyses.

### Ribonucleotides embedded in DNA at random positions induce alterations in nucleotide sequence alignment, current intensity and dwell time profiles

**Fig. 6:**
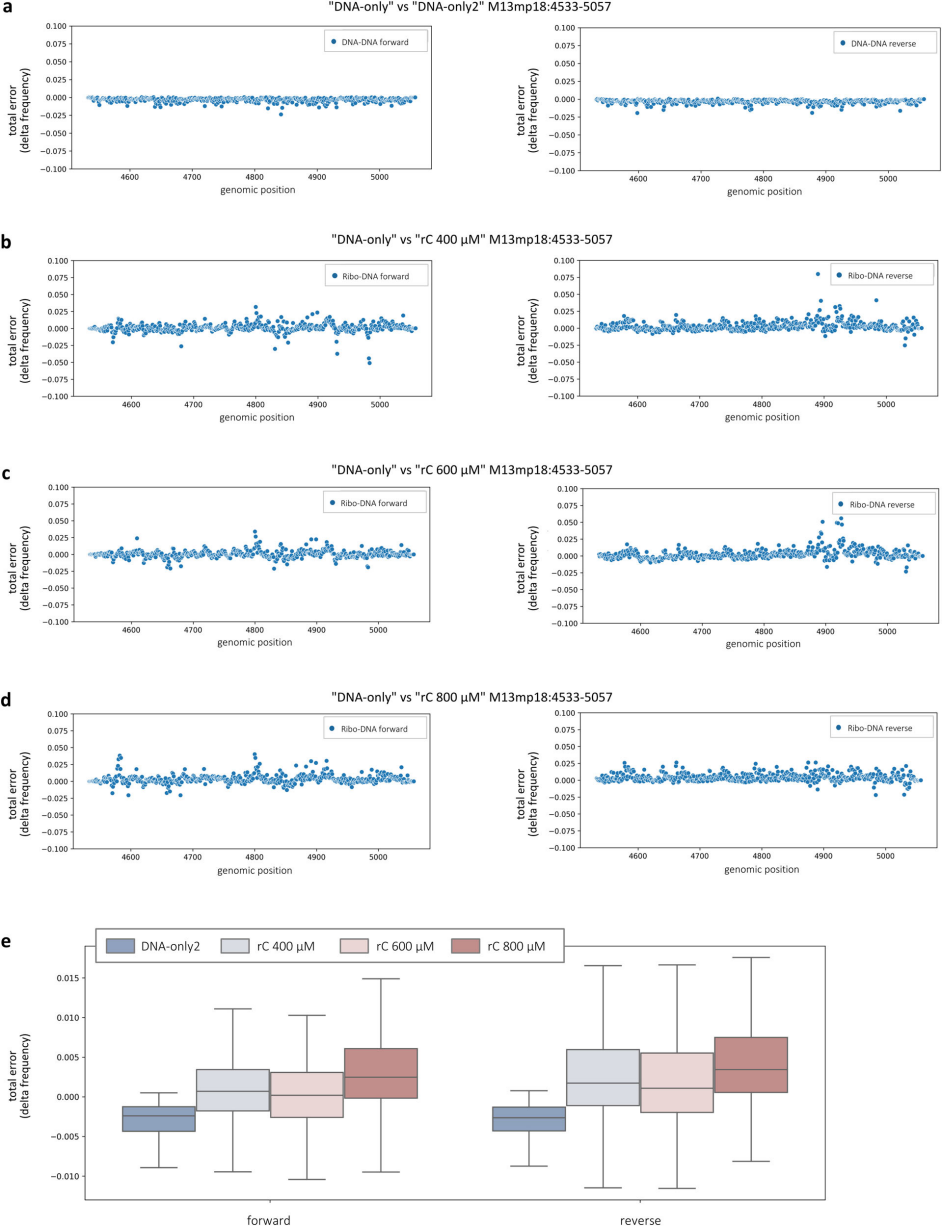
Randomly incorporated rCMPs induce alterations in nucleotide sequence alignment profiles. **a**–**d** Basecalling features were independently evaluated for forward (left panels) and reverse (right panels) strands at each M13mp18 genomic position across the 525 bp fragments containing randomly incorporated rCMPs and they were represented in graph as deltas of the total error detected (sum of mismatches and indels frequencies) at each position of each “rC” samples respect to the “DNA-only” internal control. **a** Comparison between the two DNA-only replicates. **b**–**d** Comparison between “DNA-only” and **b** “rC 400 μM”, **c** “rC 600 μM” and **d** “rC 800 μM”. **e** The same data distributions were summarised as a box plot grouped for comparison and strand. Statistical analyses were performed by two-way ANOVA (*N* = 517 genomic positions, comparison: *F*  =  249.06, d*f* = 3, *P* = 9.89e−149; strand: *F*  =  52.87, d*f* = 1, *P* = 4.23e−13; comparison/strand interaction: *F* = 8.59, d*f* = 3, *P* = 1.11e−05). The box plot shows the median as a solid black line, the first and third quartiles captured by the boundaries of the box, and whiskers defined as the first and third quartiles ± interquartile range 1.5 times.

As for the substrates containing rNMPs at defined positions, we started by evaluating the effect of randomly incorporated rCMPs on nucleotide alignment profiles. Following the pipeline described, the BAM files generated by minimap2 were split into forward (+) and reverse (-) strand sequences with respect to the M13mp18 reference. Nucleotide sequences were independently retrieved from “DNA-only”, “rC 400 μM”, “rC 600 μM” and “rC 800 μM” runs, and the nucleotide alignment profiles of forward and reverse strands were separately obtained by plotting the difference in the frequency of detection of A, C, G, T, deletions, and insertions measured at each genomic coordinate for each “rC” sample respect to the “DNA-only” control (Supplementary 3). Two different “DNA-only” technical replicates (“DNA-only” and “DNA-only2”) were compared to evaluate the basal instrumental bias (Supplementary Fig. 3a), revealing a quite “flat” distribution of the signal, indicative of consistency between the runs. When comparing “rC 400 μM” (Supplementary Fig. 3b), “rC 600 μM” (Supplementary Fig. 3c), and “rC 800 μM” (Supplementary Fig. 3d) to the internal “DNA-only” control, instead, both the forward and reverse strands showed much noisier nucleotide alignment profiles across the entire length of the substrate, consistent with the possibility of rCMP being incorporated at different positions in both strands with variable stoichiometry. To make the interpretation of results easier, basecalling features were plotted in graphs as deltas of the total error detected (sum of mismatches and indels frequencies) at each position of each “rC” sample with respect to the “DNA-only” control on the forward (Fig. 6a–d, left panels) and reverse (Fig. 6a–d, right panels) strands separately. In accordance with what is described above, the comparison between the two “DNA-only” replicates exhibited a pretty compact distribution around zero of the values of delta total error on both strands (Fig. 6a). On the other hand, the values of delta total error for internal “DNA-only” vs “rC 400 μM”, “rC 600 μM” and “rC 800 μM” on both strands, generally oscillated above or below zero (Fig. 6b–d). We subsequently collected all the values obtained from the previous analysis in a box plot in which deltas were grouped for comparison on each strand (Fig. 6e). This representation of the data allowed us to observe that the mean delta total error on both strands significantly increased together with the increasing concentration of rC from 400 to 800 μM, used in the PCR step with *Taq*-I614K (Fig. 6e). The average percentage variations on both strands respect to the internal control were +164.57%, +150.52% and +223.17% for “rC 400 μM”, “rC 600 μM” and “rC 800 μM”, respectively, with a Pearson correlation coefficient computed between rCMP concentrations and average delta total error equal to 0.90 (*p* = 0.002, divided by strand).

**Fig. 7:**
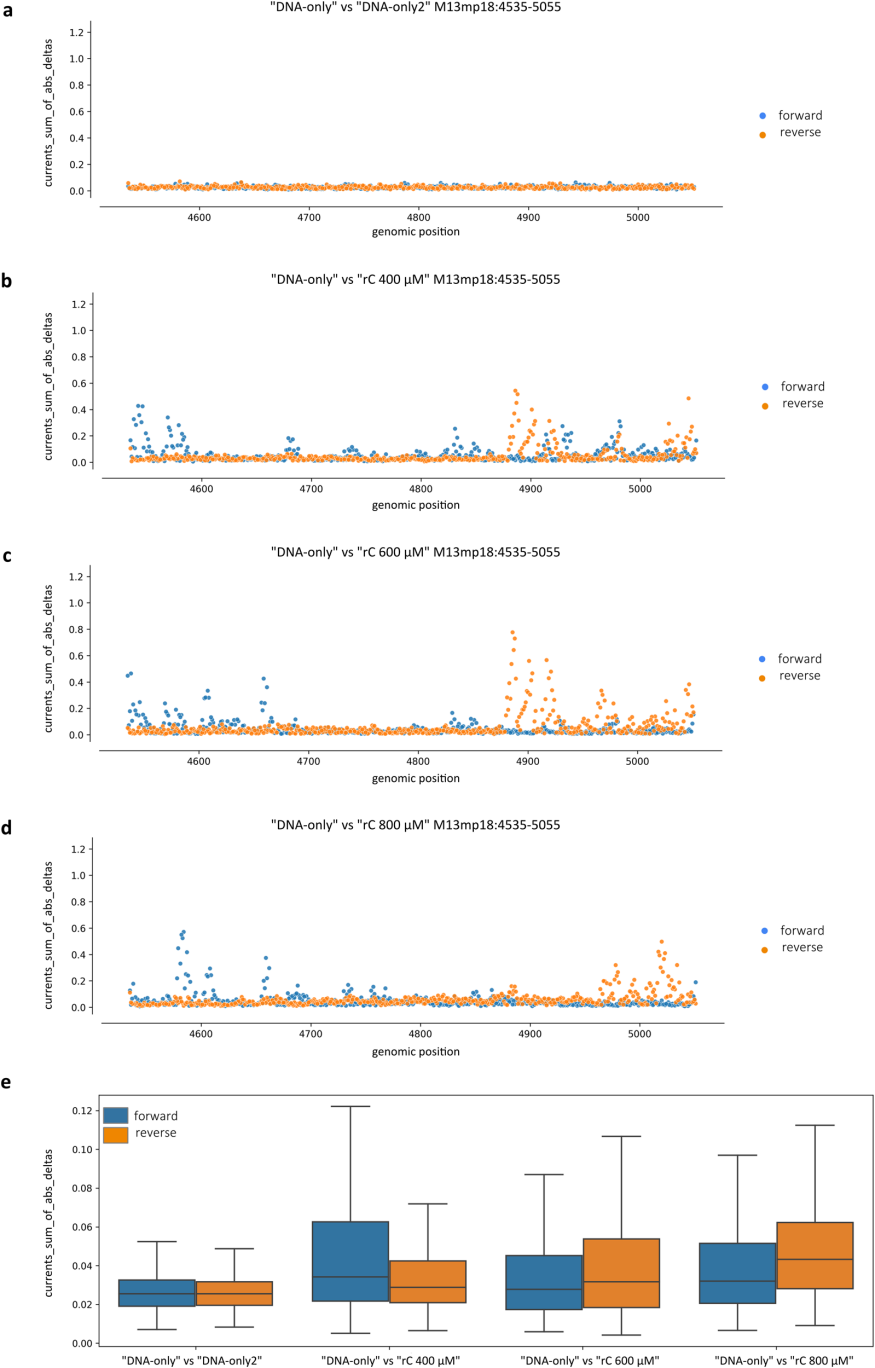
Randomly incorporated rCMPs induce alterations in current intensity profiles. **a**–**d** Ionic current values were retrieved via the f5c eventalign software. Current variations were evaluated in parallel for forward (blue) and reverse (orange) strands and computed at each M13mp18 genomic position across the 525 bp fragments containing randomly incorporated rCMPs as the sum of the absolute values of the differences between the current distributions of each “rC” sample respect to the “DNA-only” internal control. **a** Comparison between the two DNA-only replicates. **b**–**d** Comparison between “DNA-only” and **b** “rC 400 μM”, **c** “rC 600 μM” and **d** “rC 800 μM”. **e** The same data distributions were summarised as a box plot grouped for comparison and strand. Statistical analyses were performed by two-way ANOVA (*N* = 517 genomic positions, comparison: *F*  =  52.08, d*f* = 3, *P* = 4.92e−33; strand: *F*  =  6.08, d*f* = 1, *P*  = 1.37e−02; comparison/strand interaction: *F* = 7.73, d*f* = 3, *P* = 3.77e−05). The box plot shows the median as a solid black line, the first and third quartiles captured by the boundaries of the box, and whiskers defined as the first and third quartiles ± interquartile range 1.5 times.

Ionic current values were retrieved again by using the f5c eventalign software. Current variations were separately evaluated for forward and reverse strands and represented in scatter plots, where each point corresponds to a genomic coordinate, whose value was calculated as the sum of the absolute values of the delta between the current distributions of the “rC” sample respect to the “DNA-only” control (Fig. 7a–d). In line with what was observed for the basecalling features, in the scatter plot deriving from the comparison between the two “DNA-only” replicates, dots are generally very close to zero for both strands (Fig. 7a), while in the scatter plots where “rC” samples were compared to the “DNA-only” control, many points were located above the zero, reaching values between 0.6 and 0.8 (Fig. 7b–d). Data were also represented in a box plot grouped for comparison and strand, where, especially for the reverse strand, a positive correlation between rC concentrations and current perturbations is clearly observable (Fig. 7e). The average percentage variations on both strands respect to the internal control were +97.66%, +101.03% and +102.04% for “rC 400 μM”, “rC 600 μM” and “rC 800 μM”, respectively, with a high linear correlation between rCMP concentrations and average current variations with *r* = 0.82 (*p* = 0.012, divided by strand).

**Fig. 8:**
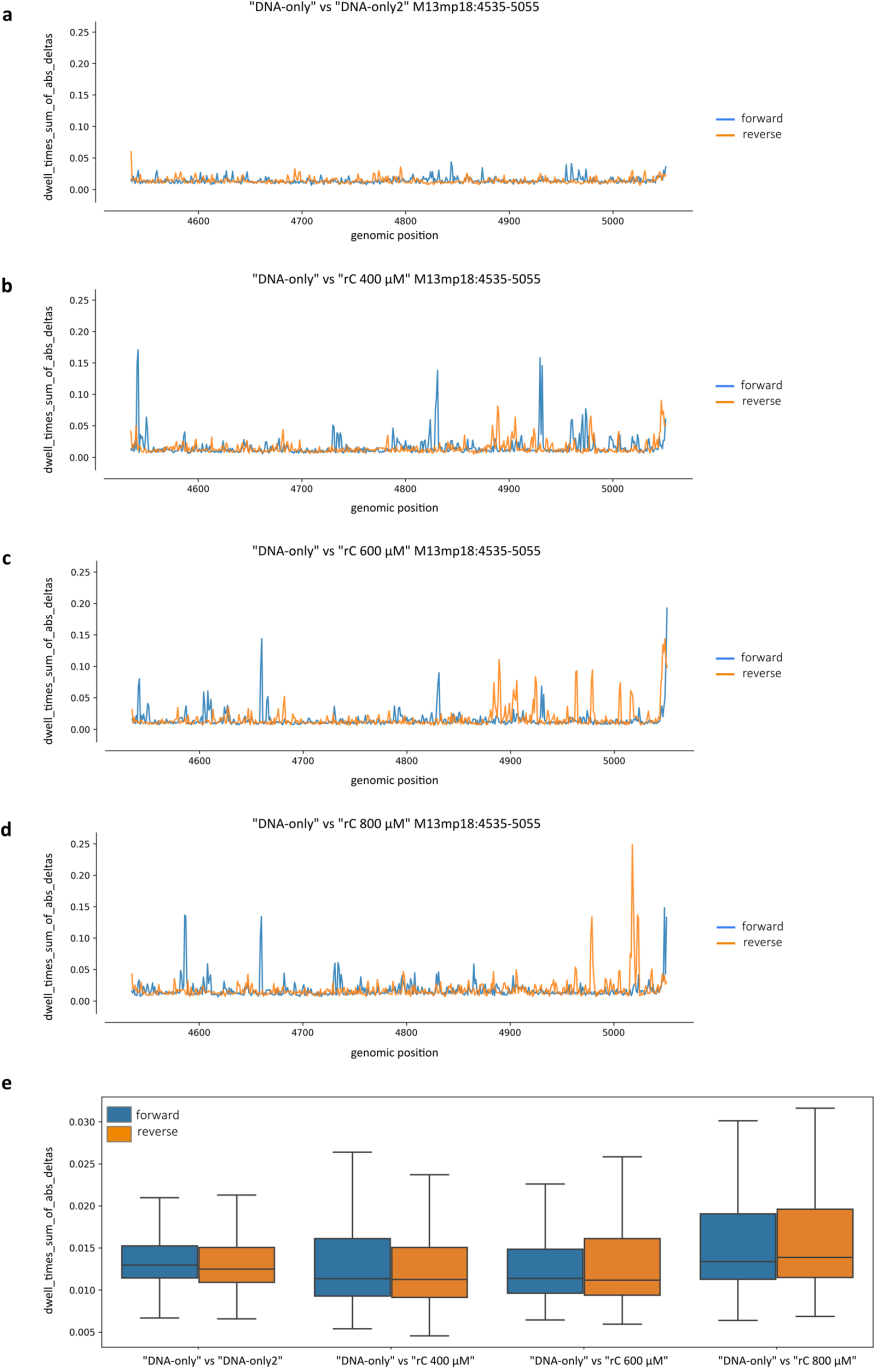
Randomly incorporated rCMPs induce alterations in dwell time profiles. **a**–**d** Dwell time values were obtained from the f5c eventalign software. Dwell time measurements were evaluated in parallel for forward (blue) and reverse (orange) strands and computed at each M13mp18 genomic position across the 525 bp fragments containing randomly incorporated rCMPs as the sum of the absolute values of the differences between the dwell time distributions of each “rC” sample respect to the “DNA-only” internal control. **a** Comparison between the two DNA-only replicates. **b**–**d** Comparison between “DNA-only” and **b** “rC 400 μM”, **c** “rC 600 μM” and **d** “rC 800 μM”. **e** The same data distributions were summarised as a box plot grouped for comparison and strand. Statistical analyses were performed by two-way ANOVA (*N* = 517 genomic positions, comparison: *F*  =  15.11, d*f* = 3, *P*  = 8.89e−10; strand: *F*  =  0.000003, d*f* = 1, *P*  = 9.98e−01; comparison/strand interaction: *F* = 7.73, d*f* = 3.29, *P* = 1.97e−02). The box plot shows the median as a solid black line, the first and third quartiles captured by the boundaries of the box, and whiskers defined as the first and third quartiles ± interquartile range 1.5 times.

Dwell times were measured with the f5c eventalign software and dwell time variations were computed similarly to what was described above. Dwell times were represented in line plots, where each position corresponding to a genomic coordinate shows the value of the sum of the absolute values of the differences between the dwell time distributions of the “rC” sample with respect to the “DNA-only” control (Fig. 8a–d). Also in this case, the analysis of “DNA-only” vs “DNA-only2” showed signal alterations close to zero in both strands (Fig. 8a), while each “rC” sample vs “DNA-only” showed an increase in the differences in dwell time distributions on both the forward and reverse strands (Fig. 8b–d). The data collected in a box plot grouped for comparison and strand showed the same behaviour already described for the previous features, confirming that also alterations in the dwell times increased together with the concentration of rC employed to obtain the substrates (Fig. 8e). The average percentage variations on both strands respect to the internal control, in this case, were lower in magnitude, being +11.16%, +14.11% and +28.98% for “rC 400 μM”, “rC 600 μM” and “rC 800 μM”, respectively, with a significant positive Pearson correlation coefficient equal to 0.87 (*p* = 0.005, divided by strand).

### Synthetic dsDNA substrates containing rCMPs at known positions or randomly distributed rCMPs show similarly perturbed profiles

The *Taq*-I614K DNA polymerase mutant is reported to incorporate 1 rCTP every 19 dCTPs in the presence of 800 μM rCTP^25^, which accounts for a probability of about 5% to have a rCMP inserted at a certain position, a value that is far below the 20% level that we analysed in the in silico washout experiments.

The analysis of “rC” samples compared to the “DNA-only” control revealed a statistically significant magnified noise in basecalling features due to raw current signals perturbations, which strictly correlated with the concentration of rCTP used for the step of PCR amplification with *Taq*-I614K (Figs. 6e, 7e and 8e), we tested the possibility to specifically recognise rCMP-related alterations at a certain position, even in a sample with such low ribonucleotide levels. We started by looking for a strategy to identify anomalous reads. To do that, we verified the existence of outliers along all the 525 reference positions by selecting all the genomic coordinates with ionic current or dwell time alterations outside a confidence interval of the mean ±2 std. dev. We then computed a general overall “anomaly” index for both features as the sum of the differences of outlier data points. In this way, we detected strong anomalies in both ionic current scores, with a fold-change for the computed indexes of +6.27, +7.22 and +5.32 for the “rC” runs with respect to the “DNA-only” run and dwell time scores with a fold-change of +0.94, +1.35 and +0.67 for “rC” samples compared to “DNA-only”. Even if these investigations indicated that the analysed features were non-uniformly affected, the presence of outliers generally appeared to be more pronounced for “rC” runs than “DNA-only” runs. We thus tested a more sophisticated approach to discern anomalous reads at a per-read level, based on an unsupervised machine learning algorithm, the Isolation Forest (iForest)^70,71^. Indeed, the identification of randomly incorporated rCMPs, which can be considered a relatively rare event occurring during the synthetic activity of *Taq*-I614K, is exactly the type of task that could easily be tackled by using models suited for the detection of anomalies like the iForest, which has been recently exploited to successfully address similar problems^72^.

**Fig. 9:**
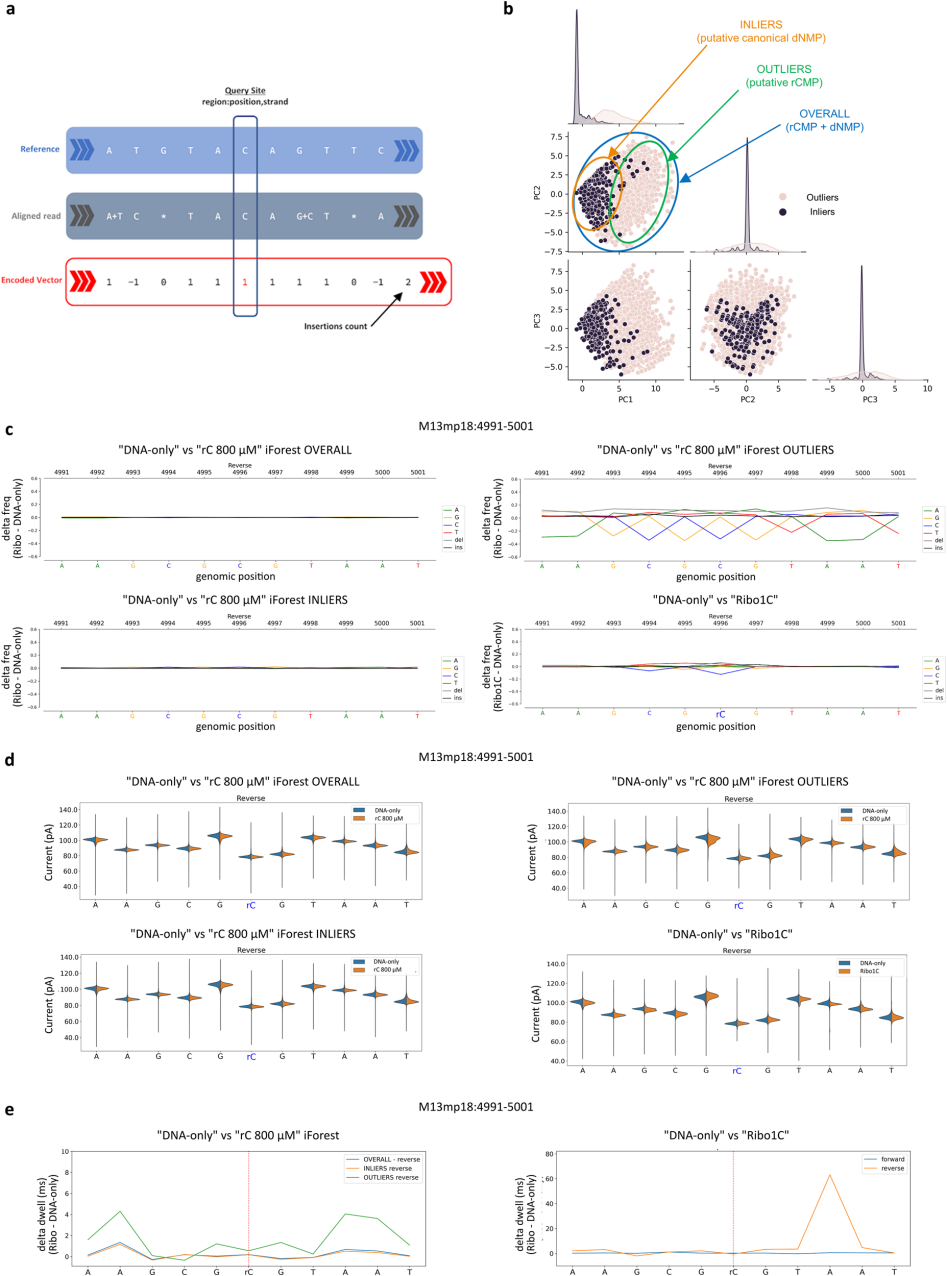
Anomalous reads isolated from the “rC 800 μM” run recapitulate the patterns of “Ribo1C” run for all the analysed features. **a** The iForest machine learning algorithm was used to classify anomalous reads from the “rC 800 μM” run. All the chunks of reads aligned onto the M13mp18:4991–5001 region, possibly containing rC at position 4996, were retrieved and encoded as one-dimensional vectors. **b** Based on the iForest inference, vectors were then classified as normal (“INLIERS”, unlikely to contain rC:4996) or anomalous (“OUTLIERS”, likely to contain rC:4996) and visualised via PCA. This labelling strategy was then exploited to classify the reads in “OVERALL”, “INLIERS” and “OUTLIERS” groups that were compared with the reads mapping on the same interval from the “Ribo1C” run, known to always include rC at position 4996. **c** Nucleotide alignment profiles, **d** current intensity profiles and **e** dwell-time profiles of all the groups.

For this investigation, we focused our attention on the “rC 800 μM” run, which was most affected, and we selected all the reads that mapped on the reverse strand and covered a ±5 nt-long region surrounding the M13mp18:4996 site, where rCMP was known to be incorporated in the previously analysed “Ribo1C” sample. In this context, a total of about 427k chunks of reads were retrieved using a custom in-house Python script, leveraging the Pysam library (see the Methods section for further details). Each read was then encoded as a 12 nt-long features vector representing the disposition of matches, mismatches and indels, as described in Fig. 9a. After the standardisation of these encoded chunks of reads, an iForest object was fit and trained using a “contamination” parameter equal to 0.05, that is roughly the expected ratio of reads containing at least one rCMP on the region of interest, according to our knowledge and expectation. We then applied the trained model to classify each read as an outlier (high anomaly score, probably carrying at least one rCMP) or an inlier (a read showing a pattern shared with the majority of the other reads, with putatively no rCMPs). A wide proportion of the variance in our dataset was due to variations between inlier and outlier reads (Fig. 9b), suggesting that the presence of a relatively low quantity of rCMPs was sufficient to generate alterations in basecalling features, distinguishable from basal instrumental noise, even if focusing on a per-read level resolution. To further confirm that the majority of the 21,310 detected outlier reads had at least one rCMP, we analysed basecalling and current features as we did for the analysis of the “Ribo1C” sample, but stratifying the reads based on the iForest prediction. We found out that the patterns of the outlier reads almost recapitulated the ones observed for the “Ribo1C” run at position 4996 corresponding to rC for all the investigated features (Fig. 9c–e), while the inlier reads appeared to be mainly unaffected. The existence of some differences between these samples can be explained by the presence of two positions in close proximity to rC:4996 (C:4994 and C:4990), where rCMP might have been incorporated by the *Taq*-I614K polymerase in some of the outlier reads, producing a more complex mixture of DNA strands.

These results indicate that the iForest model trained on alignment errors can efficiently select reads carrying rNMPs-related signals and they strongly suggest that similar machine learning models tailored for a per-read inference may be the most suitable strategy for direct identification and mapping of ribonucleotides in genomic DNA by ONT.

## Discussion

More than 10,000 and more than 1,000,000 rNMPs have been estimated to be inserted into yeast and mouse genomes, respectively, in each cell cycle, making ribonucleotides the most probable source of DNA alteration in eukaryotic cells^73^. Despite their physiological functions in specific circumstances^16,17^, the presence of chromosome-embedded ribonucleotides erroneously left in DNA is generally detrimental, as they affect DNA replication^10,12,28^ and other processes, leading to genome instability^18–20,28–30^. Different pathologies are linked to mutations in RNase H enzymes^33,37–42^, which are normally responsible for the removal of ribonucleotides from DNA^32^. It thus becomes crucial to extensively comprehend the mechanisms leading to rNMPs incorporation in chromosomes and the molecular details of ribonucleotide-induced genome instability in eukaryotic cells. To this extent, high-throughput sequencing techniques have already been developed to try to map ribonucleotides at the genomic level with single-nucleotide resolution. These methods entail an enzymatic or chemical generation of breaks at DNA–RNA junctions in genomic DNA, only allowing to indirectly infer the position of ribonucleotides in the genome. Moreover, they were applied to RNase H-depleted cells accumulating thousands of rNMPs in their template DNA, which may have altered the real sites of incorporation during a single round of DNA replication.

A tempting solution to these limitations may come from the direct sequencing strategies developed by Oxford Nanopore Technologies. Nanopore sequencing has already been successfully exploited to recognise a series of modifications both in DNA and RNA. We therefore wondered whether Nanopore sequencing could be exploited for direct identification of rNMPs embedded in DNA substrates. To evaluate the feasibility of this approach, we generated synthetic DNA molecules containing the four different ribonucleotides at known positions so that if a specific sequencing signal change was observed, it could be linked to the occurrence of a specific rNMP. Thanks to the optimisation of in-house ad hoc data analysis pipelines based on existing bioinformatic tools for ONT data collection and manipulation, we pioneered the direct detection of ribonucleotides embedded in DNA molecules by Oxford Nanopore sequencing. We assessed this either by searching for systematic, consistent, non-random “errors” in nucleotide alignments and basecalling features or by directly investigating current intensity signals and dwell times.

In order to assess Nanopore sequencing performance on samples whose characteristics were more similar to real genomes, we investigated its ability to recognise rNMPs in heterogeneous samples consisting of reads containing both rNMPs and dNMPs at a certain position. This was done initially by mixing in silico different percentages of reads containing rNMPs and dNMPs at a known position and then by analysing DNA molecules where rCMPs were randomly incorporated by PCR. Exploiting the iForest machine learning algorithm, we verified that, as for single rNMP substitutions at fixed positions, randomly incorporated rCMP was also inducing alterations in the same features previously analysed. Bimodal distributions in the current intensity profiles, similar to the ones observed in our samples, were previously demonstrated to be an effective index for the quantification of modified nucleotides in single reads in direct RNA sequencing datasets^67^. Even if our experimental set-up was still not comprehensive of all possible k-mers, our findings confirmed that alterations due to rNMPs insertions in DNA can be efficiently detected by ONT, also in a sample with low ribonucleotide levels. Therefore, our data provide a robust proof of concept that machine learning models customised to get per-read inferences may be the best approach to directly visualise and map ribonucleotides in genomic DNA through the Nanopore system. These results encourage us to build a machine learning-based model for the detection of embedded rNMPs that will require the generation of an ad-hoc comprehensive training dataset covering all the possible rNTPs incorporation contexts. Comparably to what was done in^58^, this approach will allow us to have a deeper understanding of the relevance of each used variable.

Although deoxyribonucleotides and ribonucleotides only differ in the presence of the OH group on the 2′ carbon of ribose, we showed that Nanopore sequencing can detect such small structural dissimilarity. Finding a way to increase the difference between the two types of nucleotides would possibly make the identification of rNMPs incorporated in DNA even easier.

The existence of stretches of consecutive rNMPs embedded in genomic DNA is still ambiguous, although a growing body of evidence supports the idea that they may arise from aberrations in Okazaki fragments priming or joining, R-loops formation or processing, and reparative DNA synthesis^13^. Unlike single rNMPs, which are relatively tolerated up to a certain level, rNMPs stretches were reported to be more dangerous for cell viability, aggravating replicative DNA polymerase stalling, DNA double helix distortions, and genomic instability^25,28^. The identification of multiple genomic rNMPs with the available technologies has proven to be extremely challenging. The enzymatic or chemical digestion at DNA-RNA junctions indispensable to the sequencing techniques elaborated up to now, makes it impossible to eventually discern the presence of single or multiple rNMPs at given positions in the genome. Furthermore, the most common strategies to study RNA:DNA hybrids rely on the S9.6 monoclonal antibody or on catalytically inactive RNase H1, which both indistinctly bind any type of hybrid present in the genome (R-loops, DNA replication primers, hybrids at DSBs and eventually stretches of consecutive rNMPs embedded in DNA)^13^. ONT might provide not only a solution to directly map rNMPs in chromosomes but also to distinguish sites of single and multiple ribonucleotide insertions in the genome. Demonstrating the occurrence of stretches of consecutive rNMPs embedded in eukaryotic genomes would help to clarify the contribution of the two RNase H enzymes to the recognition and processing of the different RNA substrates found in DNA, as well as the unconventional ability to synthesise multiple rNMPs insertions that certain DNA polymerases, like the Y-family polymerase η, seem to possess at least in some peculiar conditions^74–77^. This would ultimately contribute to shed light on the molecular details linking ribonucleotides, replication stress, genome instability and severe human pathologies. Naturally, the investigation of multiple ribonucleotides in DNA by Nanopore sequencing will require extra experimental and bioinformatic strategies. The raw sequencing signals deriving from multiple ribonucleotides might require a greater effort to be decoded, due to several overlapping alterations coming from each single rNMP in the stretch.

In conclusion, our work provides the first evidence that the Oxford Nanopore sequencing platform can directly distinguish ribonucleotides included in DNA molecules and the proof of concept that Nanopore sequencing may successfully be employed to directly detect and map rNMPs embedded in genomic DNA, giving further proof of the potentialities of third-generation sequencing platforms. Moreover, the basecalling alterations we detect due to embedded rNMPs may explain at least some of the basecalling errors that have been reported in ONT genomic sequencing efforts. Our work may thus contribute to helping the further development of ONT approaches.

## Methods

### Preparation of synthetic dsDNA substrates containing rNMPs at known positions

Each in vitro extension reaction was carried out with 1 μg of M13mp18 ssDNA (New England Biolabs, catalogue # N4040) and about 25 nM of complementary oligonucleotide, exploiting the Phusion™ High-Fidelity DNA Polymerase (ThermoFisher Scientific, catalogue # F530) in the presence of 200 μM of each dNTP in a final volume of 50 μL. Oligos were annealed to the template at 60 °C for 30 s and then extended by the polymerase at 72 °C for 10 min. “Ribo1A” samples were obtained starting from oligo 5′-Phosphate-AAU GGC TAT TAG TrCT TTA ATrG CGC GAA CTG ATrA GCC CT-3′, “Ribo1B” samples were obtained starting from oligo 5′-Phosphate-ArAT GGC TAT TAG TCT TTA AUG CGC GAA CTG ATA rGCC CT-3′, “Ribo1C” samples were obtained starting from oligo 5′-Phosphate-AAT GGC TAT TrAG TCT TTA ATG rCGC GAA CTG ATA GCC CT-3′, containing single rNMPs (underlined NMPs) at different positions, while “DNA-only” samples were obtained starting from oligo 5′-Phosphate-AAT GGC TAT TAG TCT TTA ATG CGC GAA CTG ATA GCC CT-3′ that does not contain rNMPs. All oligos were complementary to the M13mp18 sequence from position 4980 to 5017. Extension reaction products were then treated with the NEBNext® FFPE DNA Repair Mix (New England Biolabs, catalogue # M6630), assembling the reaction according to the “Protocol for use with Other User-supplied Library Construction Reagents” reported on the product webpage, adding 3U of T4 DNA Polymerase (New England Biolabs catalogue # M0203), and incubated at 20 °C for 3 h. The covalently closed circular dsDNA molecules obtained were O/N digested at 25 °C with MscI (New England Biolabs catalogue # R0534) and SwaI (New England Biolabs catalogue # R0604) restriction enzymes in NEB buffer 3.1. Digestion products were run on a 0.8% agarose gel, and only DNA fragments of 5545 bp were extracted from the gel and purified with the NucleoSpin Gel and PCR Clean-up kit from Macherey-Nagel, according to the manufacturer’s instructions.

### Preparation of dsDNA substrates containing randomly incorporated rNMPs

A fragment of 2026 bp was PCR-amplified from the M13mp18 ssDNA (New England Biolabs, catalogue # N4040) with forward DNA primer 5′-GAA GAA CTC AAA CTA TCG GC-3’ and reverse DNA primer 5′-GAT ATT AGC GCT CAA TTA CC-3’, by using the Phusion™ High-Fidelity DNA Polymerase (ThermoFisher Scientific, catalogue # F530) according to manufacturer’s instructions, and employed as template to PCR-amplify a fragment of 525 bp with forward DNA primer 5′-Phosphate-CCT GAA AGC GTA AGA ATA CG-3′ and reverse DNA primer 5′-Phosphate-GCC ATC ATC TGA TAA TCA GG-3′, by using the mutant *Taq*-I614K DNA Polymerase (GeneSpin Srl, www.genespin.com). The resulting 525 bp amplicon maps on the M13mp18 reference sequence at position 4533-5057, so it includes the region of the “Ribo” and “DNA-only” oligos. Reactions were carried out as described^25^ in the presence of 200 μM of each dNTP for “DNA-only” samples, and in the presence of 200 μM dATP, dGTP, and dTTP, 100 μM dCTP, and 400 μM or 600 μM, or 800 μM rCTP for “rC” samples, in a final volume of 50 μL × 48 reactions. Oligos were annealed to the template at 50 °C for 30 s and then extended by the polymerase at 72 °C for 2 min for 45 cycles. PCR products were pooled together, concentrated by precipitation in absolute ethanol with 3 M sodium acetate at pH 5.2, run on a 0.8% agarose gel, extracted from the gel, and purified with the NucleoSpin Gel and PCR Clean-up kit from Macherey–Nagel, according to manufacturer’s instructions.

### Direct DNA library preparation and sequencing

1 μg of “Ribo1A”, “Ribo1B”, “Ribo1C” or “DNA-only” dsDNA fragments obtained as described in “Preparation of synthetic dsDNA substrates containing rNMPs at known positions” were used for Nanopore libraries preparation with the SQK-LSK109 ligation sequencing kit, following the protocol “Ligation sequencing gDNA - Version: GDE_9063_v109_revAP_25May2022. Each library was loaded on a single R9.4.1 MinION flow cell using the EXP-FLP002 Flow Cell Priming Kit and a MinION Mk1B or GridION device. Two independent libraries for each of the above samples were sequenced and analysed. In total, 250 ng of “rC 400 μM”, “rC 600 μM”, “rC 800 μM” and “DNA-only” dsDNA fragments, obtained as described in “Preparation of synthetic dsDNA substrates containing randomly incorporated rNMPs”, were used for preparation of a single barcoded Nanopore library with the EXP-NBD104 native barcoding kit and the SQK-LSK109 ligation sequencing kit, following the protocol “Ligation sequencing amplicons—native barcoding—Version: NBA_9093_v109_revO_12Nov2019—Last update: 10/03/2023”. The barcoded library was loaded on two different MinION R9.4.1 flow cells using the EXP-FLP002 Flow Cell Priming Kit and a GridION device.

### Basecalling procedures and mapping

Reads were locally basecalled by Guppy 5.0.1 with GPU acceleration (NVIDIA A100-40GB) using the dna_r9.4.1_450bps_hac.cfg configuration file. The M13mp18 reference sequence was downloaded from the New England Biolabs Inc. website, and it is available at the url:

https://international.neb.com/-/media/nebus/page-images/tools-and-resources/interactive-tools/dna-sequences-and-maps/text-documents/m13mp18fsa.txt?rev=187bdc8b92314f13ba46d107b5b5553d&hash=6F212E5A79D842E6A911DF43AFAA9C07.

The fastQ files containing reads passing the Guppy quality control check, generated in the basecalling step, were merged into a single fastQ file per run and then mapped to the reference sequence by minimap2 v.2.22^78^, using standard presets and setting the -ax flag to the recommended value “map-ont”. Samtools 1.13 was used to sort, index, and filter the produced BAM files, which were then split into forward (+) and reverse (−) strand-related files using the SAM flag 16 since ribonucleotides were expected only on the reverse strand for the “Ribo1A”, “Ribo1B” and “Ribo1C” runs with single rNMPs. The same strategy was also used for the analysis of randomly incorporated rNMPs. The subsequent analysis workflow was separated into two branches conducted for both the strands of each run. The first branch aimed at retrieving and analysing the differences between “DNA-only” and “Ribo1” runs at the level of the basecalling features and nucleotide alignment profiles, the second one was focused on ionic current intensities aligned back to the reference and on related dwell times. A general overview of the workflow is schematized in Supplementary Fig. 1. For in silico simulated washout experiments, a fixed total amount of 100k reads mapping on the reverse strand were retrieved from whole BAM files from both “DNA-only” and “Ribo1A” (or “Ribo1B”, or “Ribo1C”) runs and mixed with increasing different proportions of reads containing single rNMPs. The proportions “DNA-only”:“Ribo” reads in these mixed and filtered BAM files used were 100:0, 80:20, 60:40, 40:60, 20:80 and 0:100. The generated BAM files were then deeply analysed at the level of all the features of interest. All data analysis procedures were run on HPC-HTC clusters equipped with up to 40 cores, 256 GB of RAM, and several TB of disk space.

### Alignment profiles analysis

Thanks to our in-house Python scripts, the split BAM files on forward (+) and reverse (−) strands were further investigated to retrieve their alignment profiles (basecalling features) using the Pysam software v.0.19.0 https://github.com/pysam-developers/pysam^79–81^, which leverages on the htslib C-API and the pileup engine. For each reference site where a ribonucleotide was expected, an interval covering the entire primer region (or the whole 525 bp-long PCR product for random rNMPs) was inspected and the frequencies of the aligned A, T, C, G, insertions, and deletions were retrieved. These alignment profiles from forward and reverse BAM files were analysed separately and, for the sake of clarity, alignment profiles were shown in a simplified version (Fig. 2 and S3), as differences in frequencies for each analysed feature between the two conditions/runs “DNA-only” and “Ribo”. For the in silico simulated washout experiments, mixed BAM files with increasing ratios of “Ribo” reads were investigated with the same approach focusing on the sites of interest in search for alteration due to rNMPs incorporation. For the evaluation of basecalling features alterations due to the random incorporations of rCMPs, alignment profiles were retrieved analogously and summarised as total error for every genomic position computing the sum of frequencies of unexpected aligned bases, deletions, and insertions, related to each run. So, also in this case, the difference between the total error frequencies computed on the “DNA-only” control run and the “rC” run was calculated. All data analysis procedures were run on HPC-HTC clusters equipped with up to 40 cores, 256 GB of RAM and several TB of disk space. All in-house scripts used are publicly available at https://github.com/F0nz0/nanopore-ribos.

### Current profiles analysis

For the second workflow branch, related to ionic current intensities and dwell times analysis, the f5c software v.0.7^59,82^ was exploited. The f5c software uses raw signals stored in fast5 files, together with BAM files, a reference genome sequence, and basecalled reads inside fastQ files, to detect events occurring in raw signals related to nucleotides movements inside the pore and to align these back to the reference, accordingly. Since outputs were generally very large, the extraction of events was limited to the same intervals analysed for the basecalling features via the setting of the -w flag of f5c. This allowed us to retrieve information about raw signal levels and their mapping positions with respect to each reference coordinate for a given interval. In-house Python scripts were written to pre-process these f5c events tables. In particular, following the indexing procedure required on fast5 files, the first step was to split the f5c table into two tables of events mapping either on the reverse or on the forward strand. Then, unrecognised events were filtered out and all the remaining events mapping to the same genomic position and thus belonging to the same read were collapsed together, calculating the mean current intensity value and the total dwell time. Analogously to what was done for the basecalling features, forward and reverse strand related collapsed f5c tables were used to plot and analyse ionic current intensities along with dwell times on regions around the expected ribonucleotide incorporation sites to compare “DNA-only” and “Ribo” runs. Dwell times analysis was shown as the difference of dwell times means between the two conditions (Figs. 4 and 9) when comparing control and “Ribo1” runs or as actual values (Figs. 5 and Supplementary Fig. 2) varying in function of the ratio of “Ribo1” reads used for the in silico simulated washout analysis. For the *Taq*-I614 K-related set of experiments, where the incorporation of rCMPs was expected to be random and with a varying and low stoichiometry, a different approach was used to allow the difference in currents and dwell times to emerge from the basal level of noise. F5c eventalign tables for the region of interest were retrieved and pre-processed for forward and reverse strands using the same strategy and, for each genomic position and for both ionic currents and dwell times, the sum of the absolute values of the differences between the “DNA-only” control and “rC” runs was computed and shown on the whole 525 bp-long PCR products or in an aggregated manner, stratifying per comparison and strand. All data analysis procedures were run on HPC-HTC clusters equipped with up to 40 cores, 256 GB of RAM and several TB of disk space. All in-house scripts used here are publicly available at https://github.com/F0nz0/nanopore-ribos.

### iForest clustering on the “rC 800 μM” run

For the clustering analysis, all the reads produced by sequencing “rC 800 μM—*Taq*-I614K” DNA strands mapping on a ±5 nt-long region overlapping the site M13mp18:4996 were considered. Using custom in-house Python scripts and functions, basecalling features for the iForest model were extracted from the related BAM file leveraging on the Pysam (v. 0.18) module. Aligned reads were traversed individually, and information about mismatches and indels in the surrounding interval of ±5 bases were collected and encoded using a custom vectorisation strategy (consisting of a 12-long vector), as shown in Fig. 9a, similarly to what was done for other related tasks^72^. More in detail, vectorized basecalling features were encoded for each read and for each position within the explored interval as follows: +1 for matched bases, −1 for mismatched bases and 0 for deletions, while the last integer of the vector was the insertions count. Encoded basecalling features vectors were standardised and used to fit an iForest model via the scikit-learn package and the use of the *sklearn.ensemble.IsolationForest* class, setting the maximum number of available threads (32 for our machine setup). The contamination parameter for this type of unsupervised machine learning model is of pivotal importance since it sets a threshold on the anomaly score used to classify observations as anomalies-outliers or normal ones. Based on the available literature^25^, we set the contamination parameter to 0.05 to train the model with a number of estimators equal to 200 and select all the samples with a bootstrap strategy to build individual trees. By means of our trained model, each basecalling features vector was classified as an outlier or inlier and visualised via principal component analysis (PCA) focusing on the first three principal components, subsampling a subset of inlier reads equal to the number of predicted outliers, only for visualisation purposes. In addition to PCA visualisation, the iForest predictions were used to analyse more deeply basecalling, ionic currents, and dwell-times features, separating inliers from the outliers and comparing these two groups against the “Ribo1C” reads, looking for similar patterns.

### Statistics and reproducibility

For what concerns substrates with ribonucleotides at known positions, a single “DNA-only” control library was prepared and sequenced, while two different libraries were independently prepared and sequenced for “Ribo1A”, “Ribo1B” and “Ribo1C”. The two “Ribo” replicates gave reproducible and consistent results when compared to the “DNA-only” run. In order to avoid redundancy, only results related to the comparisons between “DNA-only” and the first “Ribo” replicate were shown in Figures. Regarding substrates with randomly incorporated rCMPs, a single barcoded library was prepared and sequenced twice on two different flow cells. For each flow cell, “rC” samples were compared to the internal “DNA-only” control, giving almost identical results. As shown in Figs. 6–8, we also compared the two DNA-only technical replicates to estimate instrumental sources of variation. In order to avoid redundancy, only results related to the internal comparisons between “DNA-only” and the “rC” samples loaded on the first flow cell were shown in the Figs. When indicated in Fig. caption, statistical analysis via two-way ANOVA was performed using the Python3 stats models library. All data are available from the authors upon reasonable request.

### Reporting summary

Further information on research design is available in the Nature Portfolio Reporting Summary linked to this article.

### Supplementary information

Supplementary Information

Description of additional supplementary files

Supplementary Data 1

reporting summary

## Data Availability

FASTQ and FAST5 files can be retrieved from the SRA database at the BioProject with accession code PRJNA928310 or at the URL: https://www.ncbi.nlm.nih.gov/bioproject/PRJNA928310. The list of all SRA accession numbers, their corresponding URLs and the numeric sources of all data are available within the file Supplementary Data 1. All other data are available from the authors upon request.

## Code availability

All source code and in-house scripts used in this research work are publicly available at the GitHub repository and at 10.5281/zenodo.7709403.
