# Supplementary material for: Detection of ribonucleotides embedded in DNA by Nanopore sequencing
Source: Commun Biol. 2024 Apr 23;7:491. doi: 10.1038/s42003-024-06077-w (PMC11039623; doi:10.1038/s42003-024-06077-w)
Supplement: Supplementary file 1 — Supplementary Information [file 42003_2024_6077_MOESM1_ESM.pdf]

Supplementary Information

Detection of ribonucleotides embedded in DNA by Nanopore sequencing

Lavinia Grasso[1,§], Adriano Fonzino[2,§], Caterina Manzari[2], Tommaso Leonardi[3], Ernesto Picardi[2,4], Carmela Gissi[2,4], Federico Lazzaro[1]*, Graziano Pesole[2,4]*, and Marco Muzi-Falconi[1]*

[1] Dipartimento di Bioscienze, Università degli Studi di Milano, Via Celoria 26, 20133, Milano, Italy

[2] Dipartimento di Bioscienze, Biotecnologie e Ambiente, Università di Bari A. Moro, Via Orabona 4, 70126, Bari, Italy

[3] Center for Genomic Science of IIT@SEMM, Fondazione Istituto Italiano di Tecnologia, Via Adamello 16, 20139, Milano, Italy

[4] Istituto di Biomembrane, Bioenergetica e Biotecnologie Molecolari, Consiglio Nazionale delle Ricerche, Via Amendola 122/O, 70126, Bari, Italy

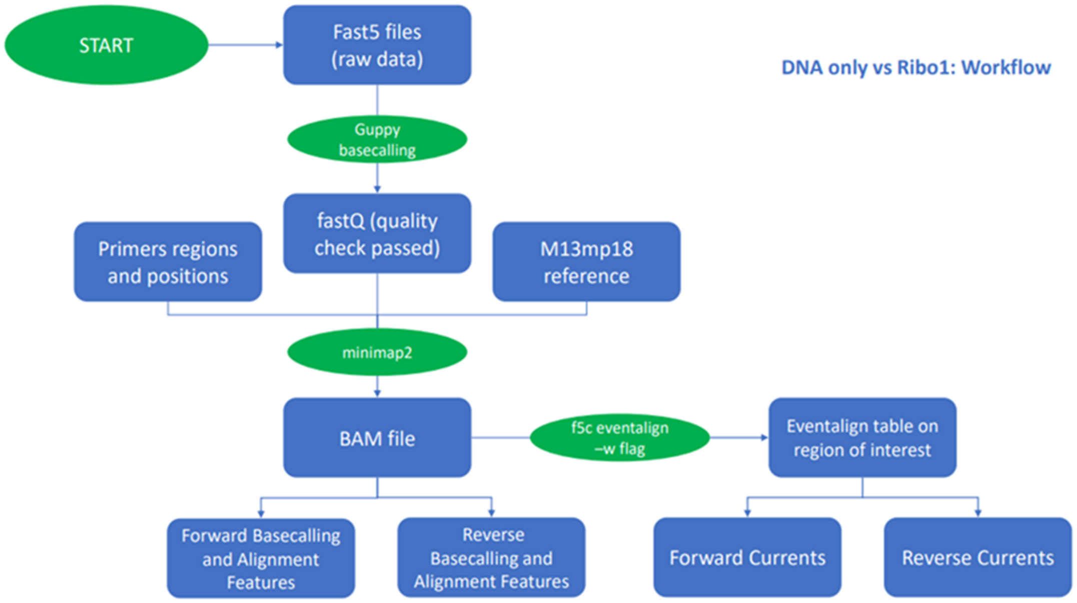

Supplementary Fig. 1. Data analysis pipeline. Workflow summary of the computational analyses performed for the identification of ribonucleotides in synthetic DNA substrates by both nucleotide alignment and current features examination. For more detailed information see the Methods section.

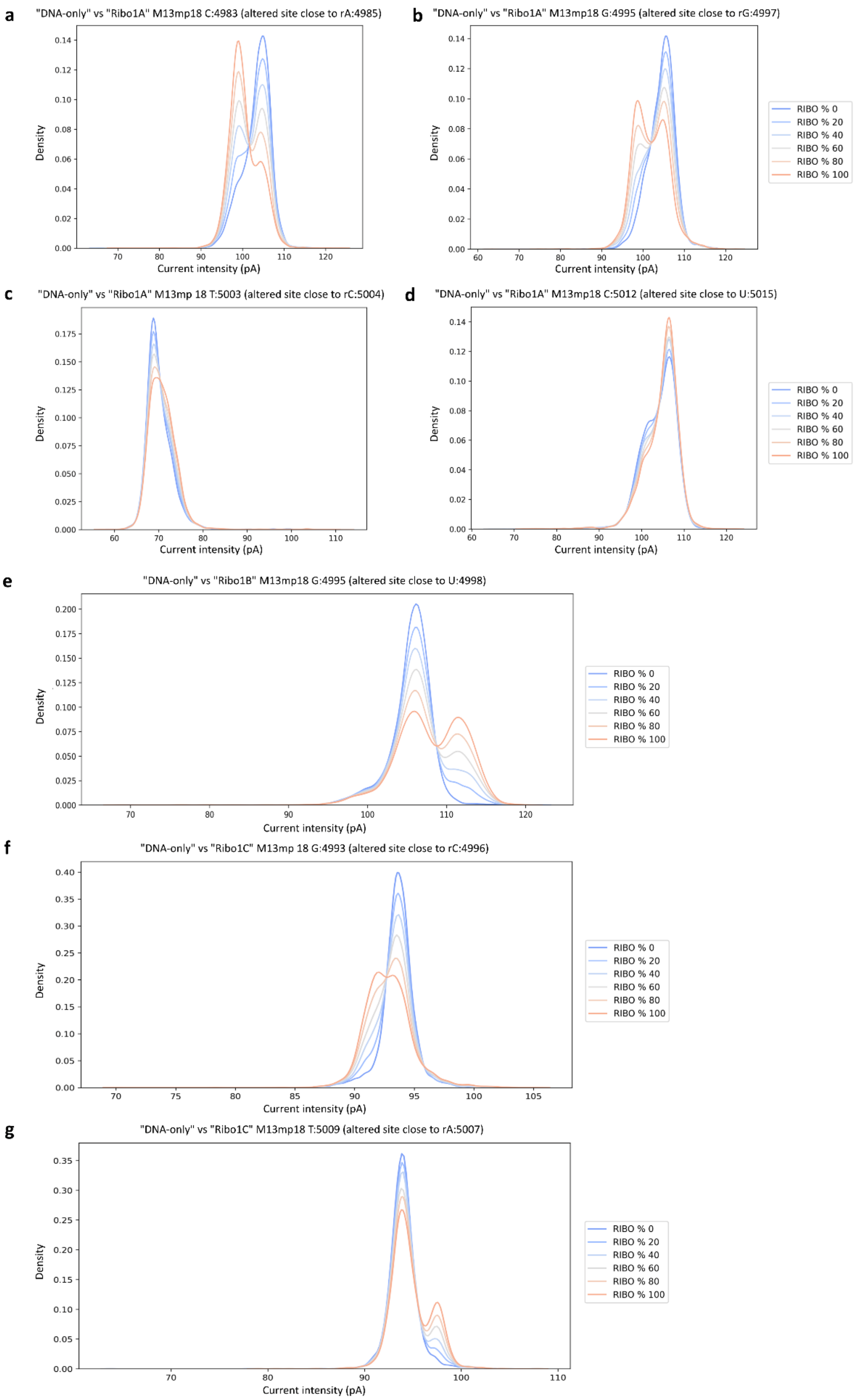
a
"DNA-only" vs "Ribo1A" M13mp18 C:4983 (altered site close to rA:4985)
Density
Current intensity (pA)
b
"DNA-only" vs "Ribo1A" M13mp18 G:4995 (altered site close to rG:4997)
Density
Current intensity (pA)
RIBO % 0
RIBO % 20
RIBO % 40
RIBO % 60
RIBO % 80
RIBO % 100
c
"DNA-only" vs "Ribo1A" M13mp 18 T:5003 (altered site close to rC:5004)
Density
Current intensity (pA)
d
"DNA-only" vs "Ribo1A" M13mp18 C:5012 (altered site close to U:5015)
Density
Current intensity (pA)
RIBO % 0
RIBO % 20
RIBO % 40
RIBO % 60
RIBO % 80
RIBO % 100
e
"DNA-only" vs "Ribo1B" M13mp18 G:4995 (altered site close to U:4998)
Density
Current intensity (pA)
RIBO % 0
RIBO % 20
RIBO % 40
RIBO % 60
RIBO % 80
RIBO % 100
f
"DNA-only" vs "Ribo1C" M13mp 18 G:4993 (altered site close to rC:4996)
Density
Current intensity (pA)
RIBO % 0
RIBO % 20
RIBO % 40
RIBO % 60
RIBO % 80
RIBO % 100
g
"DNA-only" vs "Ribo1C" M13mp18 T:5009 (altered site close to rA:5007)
Density
Current intensity (pA)
RIBO % 0
RIBO % 20
RIBO % 40
RIBO % 60
RIBO % 80
RIBO % 100

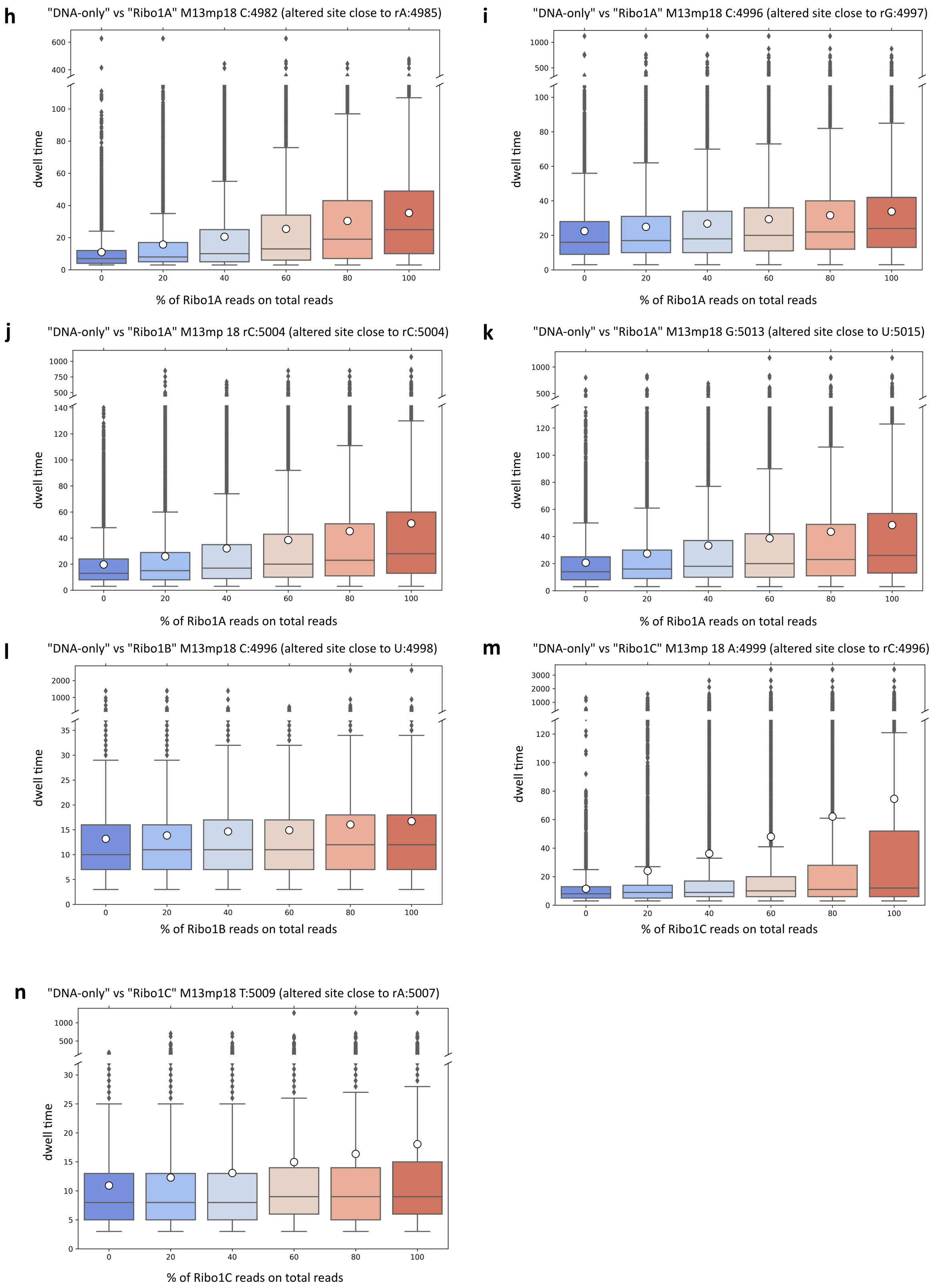

Supplementary Fig. 2. rNMPs can be recognized in a mixture of DNA and ribonucleotides-containing reads. *In-silico* simulated washout assays were performed as follows on “DNA-only”, “Ribo1A”, “Ribo1B”, and “Ribo1C” runs. A random sample made of a fixed total number of reads mapping on the reverse strand was extracted from the “DNA-only” control and the indicated “Ribo” run and mixed at different ratios of “Ribo” derived reads with respect to “DNA-only” derived reads. The effect of each ratio was deeply evaluated on altered sites within a +/- 5 nt long interval surrounding the site of rNMP incorporation. (a-g) Evaluation of the effect of different mixtures on ionic current measurements. (h-n) Evaluation of the effect of different mixtures on dwell-times. All the box plots show the median as a solid black line, the mean as a white circle, the first and third quartiles captured by the boundaries of the box, whiskers defined as the first and third quartiles ± interquartile range 1.5 times, and outliers depicted as black points. See plot titles for further details.

a

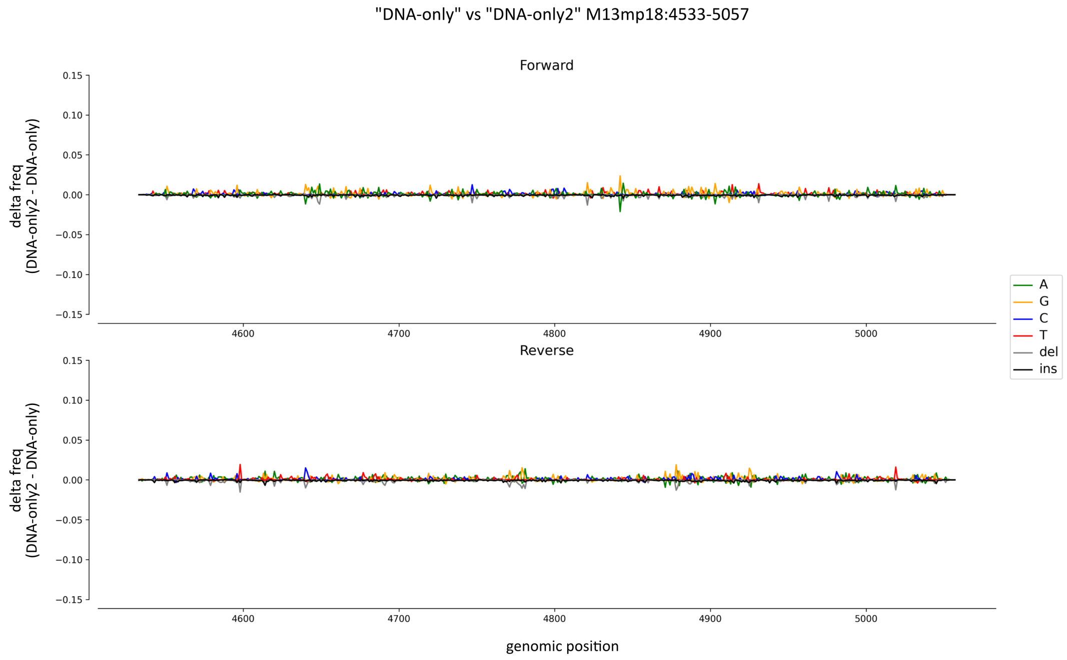
"DNA-only" vs "DNA-only2" M13mp18:4533-5057
Forward
Reverse
delta freq
(DNA-only2 - DNA-only)
0.15
0.10
0.05
0.00
−0.05
−0.10
−0.15
4600
4700
4800
4900
5000
A
G
C
T
del
ins
genomic position

b

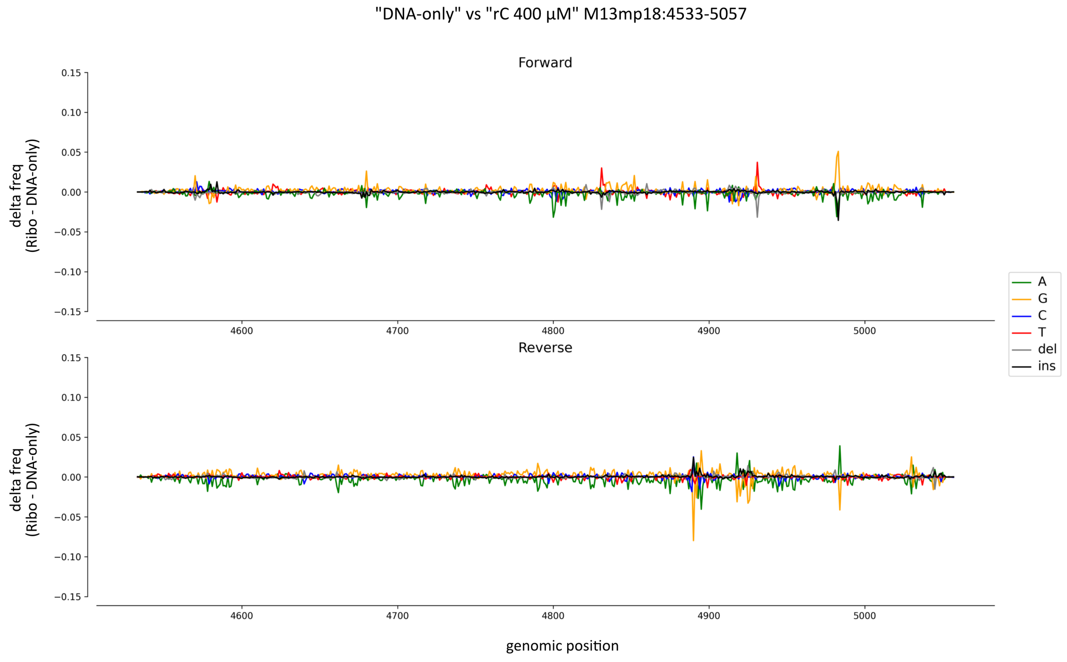
"DNA-only" vs "rC 400 µM" M13mp18:4533-5057
Forward
Reverse
delta freq
(Ribo - DNA-only)
0.15
0.10
0.05
0.00
−0.05
−0.10
−0.15
4600
4700
4800
4900
5000
A
G
C
T
del
ins
genomic position

c

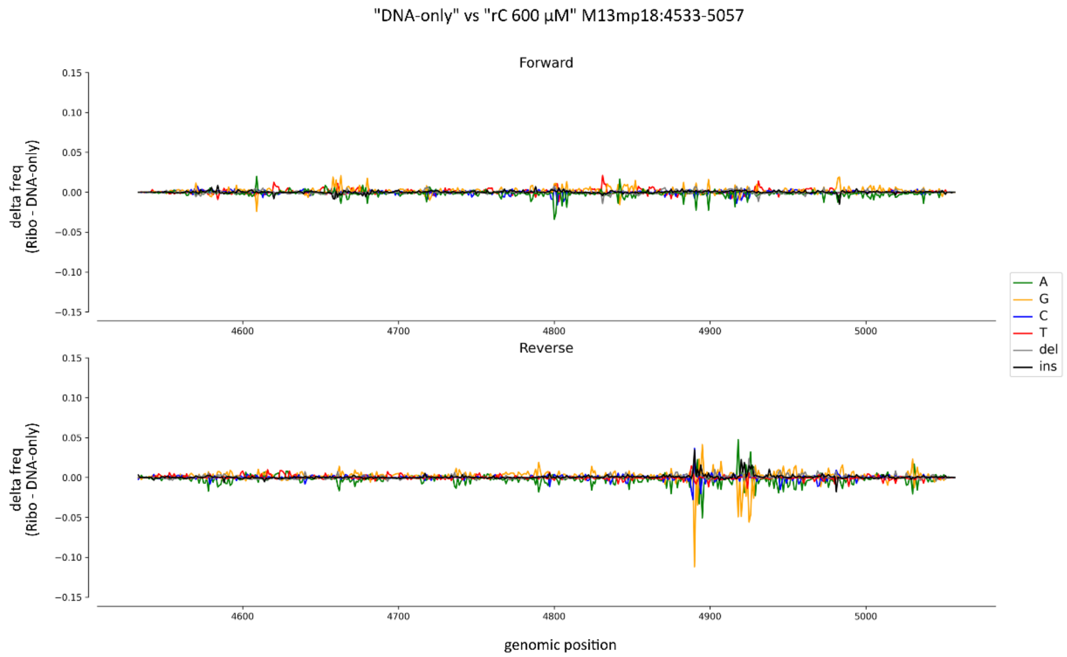

d

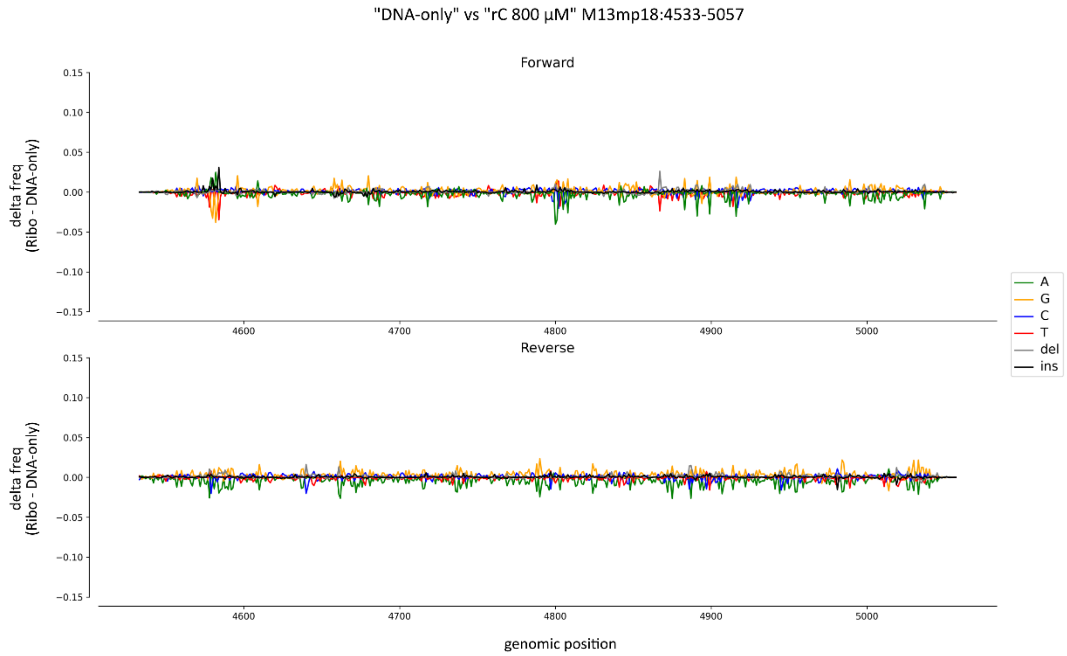

Supplementary Fig. 3. Randomly incorporated rCMPs induce alterations in nucleotide sequence alignment profiles. BAM files were split into forward and reverse strand and nucleotide sequences were independently inferred from the “DNA-only” internal control and the three “rC” samples. The difference in the frequency of detection of A (green), G (yellow), C (blue), T (red), deletions (grey), and insertions (black) was then calculated at each position of each “rC” sample compared to “DNA-only”. Each panel shows the nucleotide sequence alignment profiles of the forward (upper graphs) and reverse (bottom graphs) strands spanning the whole 525 bp-long amplicon from genomic coordinates 4533 to 5057. (A) Comparison between alignment profiles of the two “DNA-only” replicates, (B) “DNA-only” vs “rC 400 µM”, (C) “DNA-only” vs “rC 600 µM”, and (D) “DNA-only” vs “rC 800 µM”.
