# Supplementary material for: Detection of ribonucleotides embedded in DNA by Nanopore sequencing
Source: Commun Biol. 2024 Apr 23;7:491. doi: 10.1038/s42003-024-06077-w (PMC11039623; doi:10.1038/s42003-024-06077-w)
Supplement: Supplementary file 2 — Description of additional supplementary files [file 42003_2024_6077_MOESM2_ESM.docx]

Description of Additional Supplementary Files

**File name:** Supplementary Data 1

**Description:** The source data relative to the graphs in the paper
