# Supplementary material for: Detection of ribonucleotides embedded in DNA by Nanopore sequencing
Source: Commun Biol. 2024 Apr 23;7:491. doi: 10.1038/s42003-024-06077-w (PMC11039623; doi:10.1038/s42003-024-06077-w)
Supplement: Supplementary file 4 — reporting summary [file 42003_2024_6077_MOESM4_ESM.pdf]

nature portfolio

Corresponding author(s): Marco Muzi Falconi

Last updated by author(s): Mar 5, 2024

Reporting Summary

Nature Portfolio wishes to improve the reproducibility of the work that we publish. This form provides structure for consistency and transparency in reporting. For further information on Nature Portfolio policies, see our Editorial Policies and the Editorial Policy Checklist.

Statistics

For all statistical analyses, confirm that the following items are present in the figure legend, table legend, main text, or Methods section.

n/a	Confirmed	
☐	☒	The exact sample size (*n*) for each experimental group/condition, given as a discrete number and unit of measurement
☐	☒	A statement on whether measurements were taken from distinct samples or whether the same sample was measured repeatedly
☐	☒	The statistical test(s) used AND whether they are one- or two-sided<br>*Only common tests should be described solely by name; describe more complex techniques in the Methods section.*
☐	☒	A description of all covariates tested
☐	☒	A description of any assumptions or corrections, such as tests of normality and adjustment for multiple comparisons
☐	☒	A full description of the statistical parameters including central tendency (e.g. means) or other basic estimates (e.g. regression coefficient) AND variation (e.g. standard deviation) or associated estimates of uncertainty (e.g. confidence intervals)
☐	☒	For null hypothesis testing, the test statistic (e.g. *F*, *t*, *r*) with confidence intervals, effect sizes, degrees of freedom and *P* value noted<br>*Give P values as exact values whenever suitable.*
☒	☐	For Bayesian analysis, information on the choice of priors and Markov chain Monte Carlo settings
☒	☐	For hierarchical and complex designs, identification of the appropriate level for tests and full reporting of outcomes
☐	☒	Estimates of effect sizes (e.g. Cohen's *d*, Pearson's *r*), indicating how they were calculated

Our web collection on statistics for biologists contains articles on many of the points above.

Software and code

Policy information about availability of computer code

Data collection	Nanopore sequencing reads were basecalled with Guppy 5.0.1
Data analysis	Guppy 5.0.1 was used to basecall the reads<br>Minimap2 v.2.22 was used to align the fastq reads against the reference genome<br>Samtools 1.13 was used to retrieve basecalling features and to manipulate alignment files<br>F5c v.0.7 was used to resquiggle raw currents signal against the reference sequence<br>Python 3.8 was used to retrieve basecalling features inside the Python code<br>Matploylib.pyplot and Seaborn were used to produce plots and figures<br>All source code and in-house scripts used in this research work are publicly available at the GitHub repository https://github.com/F0nz0/nanopore-ribos and at DOI: 10.5281/zenodo.7709403

For manuscripts utilizing custom algorithms or software that are central to the research but not yet described in published literature, software must be made available to editors and reviewers. We strongly encourage code deposition in a community repository (e.g. GitHub). See the Nature Portfolio guidelines for submitting code & software for further information.

Data

Policy information about availability of data

All manuscripts must include a data availability statement. This statement should provide the following information, where applicable:

- Accession codes, unique identifiers, or web links for publicly available datasets
- A description of any restrictions on data availability
- For clinical datasets or third party data, please ensure that the statement adheres to our policy

FASTQ and FAST5 files can be retrieved from the SRA database at the BioProject with accession code PRJNA928310 or at the URL: https://www.ncbi.nlm.nih.gov/bioproject/PRJNA928310. The list of all SRA accession numbers, their corresponding URLs and the numeric sources of all data are available within the file Supplementary Data 1. All other data are available from the authors upon request.
The genomic reference sequence was downloaded from the New England Biolabs Inc. website, and it is available at the url: https://international.neb.com/-/media/nebus/page-images/tools-and-resources/interactive-tools/dna- sequences-and-maps/text- documents/m13mp18fsa.txt?rev=187bdc8b92314f13ba46d107b5b5553d&hash=6F212E5A79D842E 6A911DF43AFAA9C07.

Research involving human participants, their data, or biological material

Policy information about studies with human participants or human data. See also policy information about sex, gender (identity/presentation), and sexual orientation and race, ethnicity and racism.

Reporting on sex and gender	no human data used
Reporting on race, ethnicity, or other socially relevant groupings	no human data used
Population characteristics	no human data used
Recruitment	no human data used
Ethics oversight	no human data used

Note that full information on the approval of the study protocol must also be provided in the manuscript.

Field-specific reporting

Please select the one below that is the best fit for your research. If you are not sure, read the appropriate sections before making your selection.

☒ Life sciences ☐ Behavioural & social sciences ☐ Ecological, evolutionary & environmental sciences

For a reference copy of the document with all sections, see nature.com/documents/nr-reporting-summary-flat.pdf

Life sciences study design

All studies must disclose on these points even when the disclosure is negative.

Sample size	No sample size calculation was required
Data exclusions	No data were excluded from the analysis
Replication	Two Technical replicates were performed
Randomization	No randomization performed
Blinding	We did not work blind since to test the system we need to know the coordinates of the expected incorporation sites or the type of incorporation.

Reporting for specific materials, systems and methods

We require information from authors about some types of materials, experimental systems and methods used in many studies. Here, indicate whether each material, system or method listed is relevant to your study. If you are not sure if a list item applies to your research, read the appropriate section before selecting a response.

Materials & experimental systems

n/a	Involved in the study
☒	☐ Antibodies
☒	☐ Eukaryotic cell lines
☒	☐ Palaeontology and archaeology
☒	☐ Animals and other organisms
☒	☐ Clinical data
☒	☐ Dual use research of concern
☒	☐ Plants

Methods

n/a	Involved in the study
☒	☐ ChIP-seq
☒	☐ Flow cytometry
☒	☐ MRI-based neuroimaging

Plants

Seed stocks	na
Novel plant genotypes	na
Authentication	na
